# Differentiation-Dependent KLF4 Expression Promotes Lytic Epstein-Barr Virus Infection in Epithelial Cells

**DOI:** 10.1371/journal.ppat.1005195

**Published:** 2015-10-02

**Authors:** Dhananjay M. Nawandar, Anqi Wang, Kathleen Makielski, Denis Lee, Shidong Ma, Elizabeth Barlow, Jessica Reusch, Ru Jiang, Coral K. Wille, Deborah Greenspan, John S. Greenspan, Janet E. Mertz, Lindsey Hutt-Fletcher, Eric C. Johannsen, Paul F. Lambert, Shannon C. Kenney

**Affiliations:** 1 McArdle Laboratory for Cancer Research, University of Wisconsin School of Medicine and Public Health, Madison, Wisconsin, United States of America; 2 Cellular and Molecular Biology Graduate Program, University of Wisconsin School of Medicine and Public Health, Madison, Wisconsin, United States of America; 3 Department of Microbiology and Immunology, Center for Molecular and Tumor Virology and Feist-Weiller Cancer Center, Louisiana State University Health Sciences Center, Shreveport, Louisiana, United States of America; 4 Medical Microbiology and Immunology Graduate Program, University of Wisconsin School of Medicine and Public Health, Madison, Wisconsin, United States of America; 5 Department of Orofacial Sciences, School of Dentistry, University of California, San Francisco, San Francisco, California, United States of America; 6 Department of Medicine, University of Wisconsin School of Medicine and Public Health, Madison, Wisconsin, United States of America; Emory University, UNITED STATES

## Abstract

Epstein-Barr virus (EBV) is a human herpesvirus associated with B-cell and epithelial cell malignancies. EBV lytically infects normal differentiated oral epithelial cells, where it causes a tongue lesion known as oral hairy leukoplakia (OHL) in immunosuppressed patients. However, the cellular mechanism(s) that enable EBV to establish exclusively lytic infection in normal differentiated oral epithelial cells are not currently understood. Here we show that a cellular transcription factor known to promote epithelial cell differentiation, KLF4, induces differentiation-dependent lytic EBV infection by binding to and activating the two EBV immediate-early gene (BZLF1 and BRLF1) promoters. We demonstrate that latently EBV-infected, telomerase-immortalized normal oral keratinocyte (NOKs) cells undergo lytic viral reactivation confined to the more differentiated cell layers in organotypic raft culture. Furthermore, we show that endogenous KLF4 expression is required for efficient lytic viral reactivation in response to phorbol ester and sodium butyrate treatment in several different EBV-infected epithelial cell lines, and that the combination of KLF4 and another differentiation-dependent cellular transcription factor, BLIMP1, is highly synergistic for inducing lytic EBV infection. We confirm that both KLF4 and BLIMP1 are expressed in differentiated, but not undifferentiated, epithelial cells in normal tongue tissue, and show that KLF4 and BLIMP1 are both expressed in a patient-derived OHL lesion. In contrast, KLF4 protein is not detectably expressed in B cells, where EBV normally enters latent infection, although KLF4 over-expression is sufficient to induce lytic EBV reactivation in Burkitt lymphoma cells. Thus, KLF4, together with BLIMP1, plays a critical role in mediating lytic EBV reactivation in epithelial cells.

## Author Summary

Lytic EBV infection of differentiated oral epithelial cells results in the release of infectious viral particles and is required for efficient transmission of EBV from host to host. Lytic infection also causes a tongue lesion known as oral hairy leukoplakia (OHL). However, surprisingly little is known in regard to how EBV gene expression is regulated in epithelial cells. Using a stably EBV- infected, telomerase-immortalized normal oral keratinocyte cell line, we show here that undifferentiated basal epithelial cells support latent EBV infection, while differentiation of epithelial cells promotes lytic reactivation. Furthermore, we demonstrate that the KLF4 cellular transcription factor, which is required for normal epithelial cell differentiation and is expressed in differentiated, but not undifferentiated, normal epithelial cells, induces lytic EBV reactivation by activating transcription from the two EBV immediate-early gene promoters. We also show that the combination of KLF4 and another differentiation-dependent cellular transcription factor, BLIMP1, synergistically activates lytic gene expression in epithelial cells. We confirm that KLF4 and BLIMP1 expression in normal tongue epithelium is confined to differentiated cells, and that KLF4 and BLIMP1 are expressed in a patient-derived OHL tongue lesion. These results suggest that differentiation-dependent expression of KLF4 and BLIMP1 in epithelial cells promotes lytic EBV infection.

## Introduction

Epstein-Barr Virus (EBV) is a human gamma-herpesvirus that causes the clinical syndrome infectious mononucleosis [1], and contributes to several types of human malignancy. EBV, which primarily infects B cells and oropharyngeal epithelial cells, is associated with the development of both B cell and epithelial cell tumors in humans, including Burkitt lymphoma, Hodgkin Disease, nasopharyngeal carcinoma (NPC) and gastric carcinoma [2,3]. Like all herpesviruses, EBV undergoes both latent and lytic forms of infection in normal cells, and both types of infection are essential for the long-term success of the virus. However, EBV-infected tumors primarily contain cells with latent viral infection, since this type of infection allows expression of the major viral transforming proteins but does not cause virally-mediated cell killing [2,4].

In contrast to B cells, relatively little is known about the regulation of EBV infection in normal epithelial cells. The memory B cell compartment serves as the major reservoir for life-long latent EBV infection in humans [5]. EBV-infected B cells can be reactivated to the lytic form of infection, which is required for production of infectious viral particles, following strong B cell receptor (BCR) stimulation and/or plasma cell differentiation [4,6–8]. Normal (untransformed) oropharyngeal epithelial cells also support the lytic form of EBV infection [9–11], but there is currently little evidence that these cells can undergo persistent latent infection. Long-term latent EBV persistence following infection of telomerase-immortalized nasopharygeal epithelial cells has been reported to require over-expression of the oncogene, cyclin D1, as well as repression of the p16 tumor suppressor protein [12]. Thus, the ability of EBV to establish long-term latency in epithelial cells *in vitro* may require that the cells already be abnormal.

Much of our current knowledge regarding EBV infection of untransformed epithelium in humans is derived from papers examining EBV gene/protein expression in oral hairy leukoplakia (OHL) lesions of immunosuppressed patients [10,13,14]. These studies have suggested that EBV infection in OHL lesions is limited to the more differentiated layers of the tongue epithelium, and is completely lytic. Consistent with these findings, a recent *in vitro* study examining EBV infection in normal stratified oral epithelial cells grown in organotypic raft culture found completely lytic EBV infection in the differentiated cell layers, but no evidence of latent or lytic infection in undifferentiated basal cells [15].

Whether epithelial cell differentiation promotes lytic EBV reactivation and/or whether EBV can even infect normal undifferentiated epithelial cells in humans is not totally clear. It remains possible that EBV (similar to human papilloma virus [HPV])[16,17] maintains persistent latent infection in a small number of undifferentiated normal basal epithelial cells and converts to productive viral infection during epithelial cell differentiation. Studies done by several groups *in vitro* have suggested a correlation between epithelial cell differentiation of EBV-infected carcinoma lines and EBV lytic reactivation, but the methods used to induce differentiation in these studies have multiple different effects and thus may have activated lytic EBV gene expression through mechanism(s) distinct from differentiation *per se* [18–21]. Ideally, the effect of differentiation on EBV gene expression should be examined using air-interface organotypic raft cultures, as has previously been done in HPV-infected cell lines [22]. However, such studies have been limited by the availability of a long-term EBV infected epithelial cell line that retains the ability to undergo differentiation using this technique.

The lytic viral cascade in latently EBV-infected cells is initiated by expression of the two viral immediate-early (IE) genes, BZLF1 (Z) and BRLF1 (R), that encode the transcription factors Z and R respectively [23–30]. The Z and R proteins initially activate each other’s promoters and then cooperate to induce expression of the entire cadre of lytic genes required for lytic viral DNA replication and virion production [28,31–35]. While overexpression of Z alone is sufficient to induce EBV lytic reactivation in essentially all previously examined latently EBV-infected cell lines, we recently identified an EBV-infected cell line (EBV-infected telomerase-immortalized normal oral keratinocytes, referred to here as NOKs-Akata cells) in which only R, and not Z, overexpression can induce lytic viral reactivation [36]. These results suggest that EBV lytic reactivation in normal epithelial cells, in contrast to EBV-infected carcinoma cells and EBV-infected B cells, may be more dependent upon cellular transcription factors that activate the viral IE promoter (Rp) driving R transcription rather than the IE promoter (Zp) driving Z transcription. We recently showed that BLIMP1, a cellular transcription factor whose expression is greatly enhanced by both B cell and epithelial cell differentiation [37,38], can activate Rp in reporter gene assays and reactivate low-level lytic gene EBV expression in a subset of EBV-infected cell lines [39]. However, the cellular factors that activate Rp (and potentially Zp) during epithelial cell differentiation remain largely unexplored.

In this paper, we have used organotypic raft cultures of NOKs-Akata cells to demonstrate that epithelial cell differentiation of latently infected cells induces lytic EBV reactivation. Furthermore, we show that a differentiation-dependent cellular transcription factor, KLF4 [40,41], binds to and activates both the Zp and Rp EBV promoters, and collaborates with BLIMP1 to synergistically induce high-level lytic EBV reactivation in latently infected epithelial cells. We also find that KLF4 and BLIMP1 are only expressed within differentiated cells in normal tongue epithelium, and that epithelial cells in a patient-derived OHL lesion express both KLF4 and BLIMP1. In contrast, we do not detect KLF4 protein in either EBV-infected or uninfected B cells. We propose that the increased expression of KLF4 and BLIMP1 that occurs during normal epithelial cell differentiation promotes lytic EBV infection. Conversely, the lack of both KLF4 and BLIMP1 expression in normal undifferentiated epithelial cells, undifferentiated NPC tumors, and B cells promotes viral latency.

## Results

### Lytic EBV protein expression in NOKs-Akata cells is restricted to the more differentiated cell layers in raft culture

**Fig 1:**
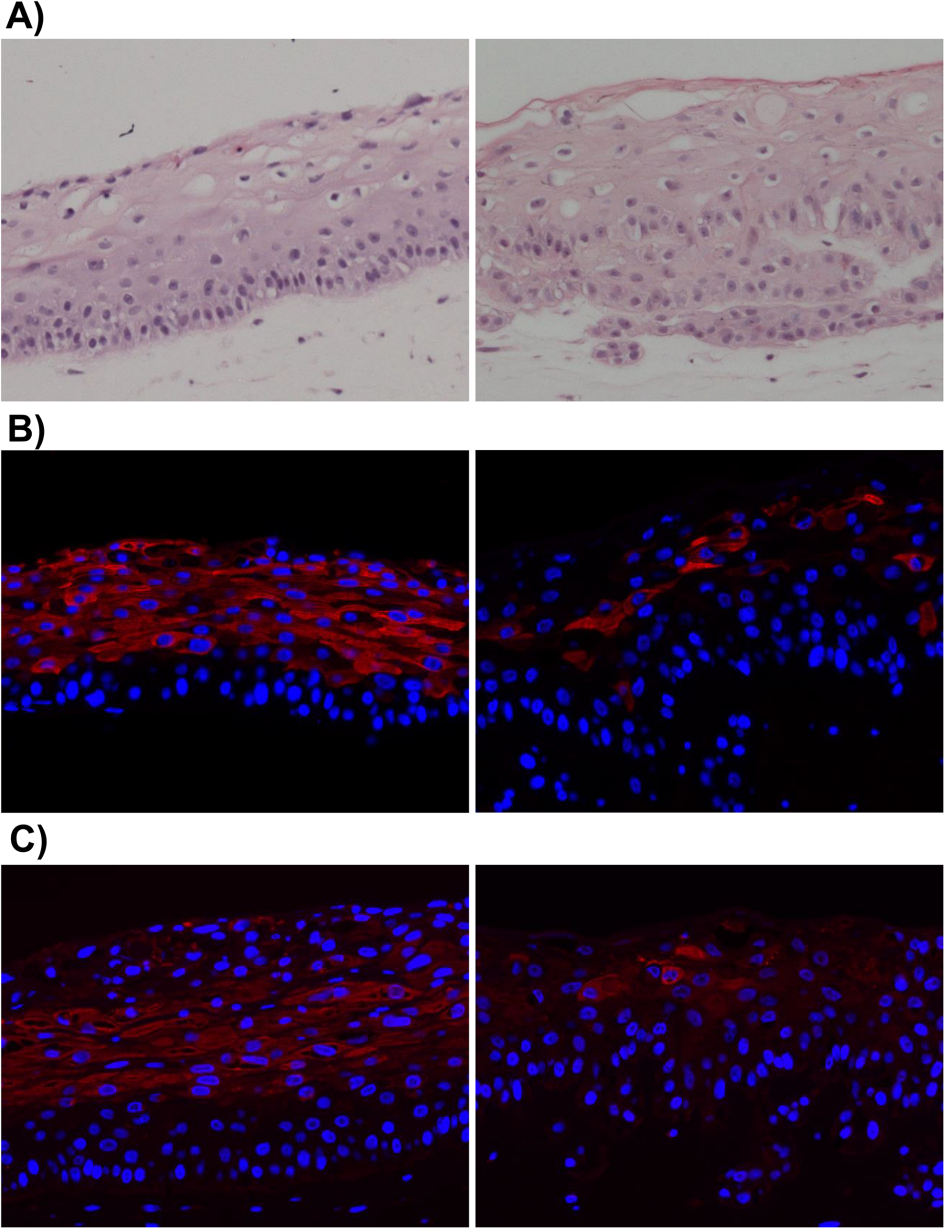
EBV-infected NOKs cells retain ability to be partially differentiated. Uninfected NOKs (left panel) and EBV-infected NOKs-Akata (right panel) cells were grown in organotypic raft culture, samples formalin fixed, embedded in paraffin and 5-micron thick sections analyzed. A). Images from hematoxylin and eosin (H&E) stained sections are shown. Note the presence of squames at the top of the NOKs-Akata raft indicative of terminal differentiation. Also note that the basal compartment of the NOKs-Akata raft is much less well organized with signs of migration/invasion of the epithelial cells into the underlying dermal equivalent (poorly stained portion of the image) at the bottom of the image. Immunofluorescent images from sections of rafts stained with antibody against the epithelial cell differentiation markers, cytokeratin 10 (B), and involucrin (C) are shown. Cytokeratin 10 (K10)- and involucrin- positive cells are stained red. Blue nuclear counter stain is DAPI. Note the paucity of K10- and involucrin- positive cells in the NOKs-Akata raft indicating a perturbation of normal differentiation by EBV, even though morphologically terminal differentiation does occur as evidenced by the presence of squames.

The differentiation program of stratified epithelia can be recapitulated *in vitro* using the organotypic culture technique in which epithelial cells are plated on a dermal equivalent composed of human fibroblasts embedded in collagen, allowed to grow to confluence, then raised to the air/liquid interface (thereby commonly referred to as 'raft' cultures) and cultured for approximately two weeks’ time during which the epithelial cells proliferate, stratify, and daughter cells that lose contact with the dermal equivalent undergo terminal differentiation. Rafting is considered the gold standard method for recapitulating the normal differentiation program of stratified epithelial *in vitro*. To determine if EBV-infected NOKs cells (NOKs-Akata) retain the ability to be differentiated, uninfected and EBV-infected NOKs were grown in raft cultures. As shown in Fig 1A, the EBV-infected NOKs cells undergo stratification as does the uninfected parental cell population, with classical morphological signs of differentiation including the presence of terminally differentiated squames at the top surface, clearly visible in the EBV-infected NOKs. Closer examination of the EBV infected cells, however, showed evidence of a much less well organized basal layer of epithelial cells (the layer of cells directly in contact with the underlying dermal equivalent), with signs of epithelial migration/invasion into the underlying dermal equivalent, suggestive of a partial disruption in the normal program of epithelial cell differentiation. Consistent with these morphological changes, the pattern of expression of cytokeratin 10 (K10) and involucrin, cellular proteins whose expression is induced in suprabasal cells, was less uniform in the EBV-infected raft culture (Fig 1B and Fig 1C). These observations validate recent findings made using other approaches for inducing epithelial cell differentiation that less well recapitulate the *in vivo* process: suspension of epithelial cells in methylcellulose or growth of epithelial cells in high concentration of calcium chloride [42].

**Fig 2:**
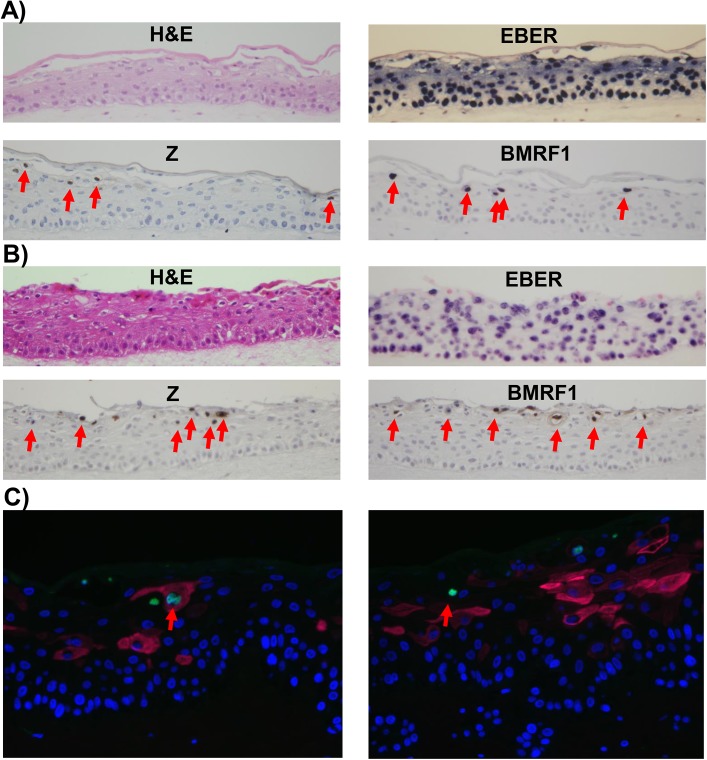
Lytic EBV protein expression in NOKs-Akata cells is restricted to the more differentiated cell layers. Two different independently generated NOKs-Akata cell lines (panels A and B) were grown in organotypic air-interface raft culture, and *in situ* hybridization or immunohistochemistry was performed to detect expression of the EBV EBERs or lytic EBV proteins (Z and BMRF1) as indicated. Examples of Z and BMRF1 stained cells are indicated by red arrows. C) NOKs-Akata cells grown in organotypic air-interface raft culture were examined by immunofluorescence using both anti-K10 (red) and anti-Z (green) antibodies. An example of a Z and K10 co-staining cell is shown in the left panel, and a Z-positive/K10 negative cell is shown in the right panel.

We next examined whether signs of lytic EBV reactivation arise within raft cultures of two independently isolated populations of EBV-infected NOKs (Fig 2A and 2B). First we stained for EBV-encoded small nuclear non-coding RNAs (EBERs), which are highly expressed in latently EBV-infected cells, to confirm that the raft cultures retained EBV. EBERs were detected by *in situ* hybridization throughout the raft cultures of EBV-infected NOKs, while uninfected cells had no detectable EBERs (S1 Fig). Next we stained for two markers of lytic reactivation, the EBV immediate-early BZLF1 gene encoding the Z protein and the early lytic BMRF1 gene encoding the viral DNA polymerase processivity factor. Cells positive for these markers were exclusively detected within the suprabasal compartment of the raft culture. Uninfected NOKs cells did not stain positively (S1 Fig). Immunofluorescence co-staining using antibodies directed against K10 and Z showed that while expression of Z can be associated with expression of differentiation marker K10 (Fig 2C, left panel), some Z-positive cells do not express K10 (Fig 2C, right panel).

To determine if epithelial cell differentiation is associated with EBV genome amplification, fluorescence *in situ* hybridization (FISH) assays were performed using a probe that detects EBV genome DNA. While small green foci representing latent EBV genomes were present in every cell, cells with highly positive signals for the EBV genome (due to lytically-infected EBV) were only detected in the more differentiated cell layers (S2 Fig). Together, these results indicate that expression of lytic EBV genes and lytic viral DNA replication arises preferentially within the differentiating, suprabasal compartment, indicating that lytic reactivation arises when epithelial cells are triggered to undergo terminal differentiation, even though that differentiation is partially perturbed by EBV. The relative rarity of lytically infected cells in rafted NOKs-Akata cells in comparison to EBV-infected rafted primary oral epithelial cells [15] may reflect the decreased differentiation in NOKs-Akata cells.

### Treatment of NOKs-Akata cells with the differentiating agents, TPA and calcium chloride/FBS, also results in lytic EBV reactivation

**Fig 3:**
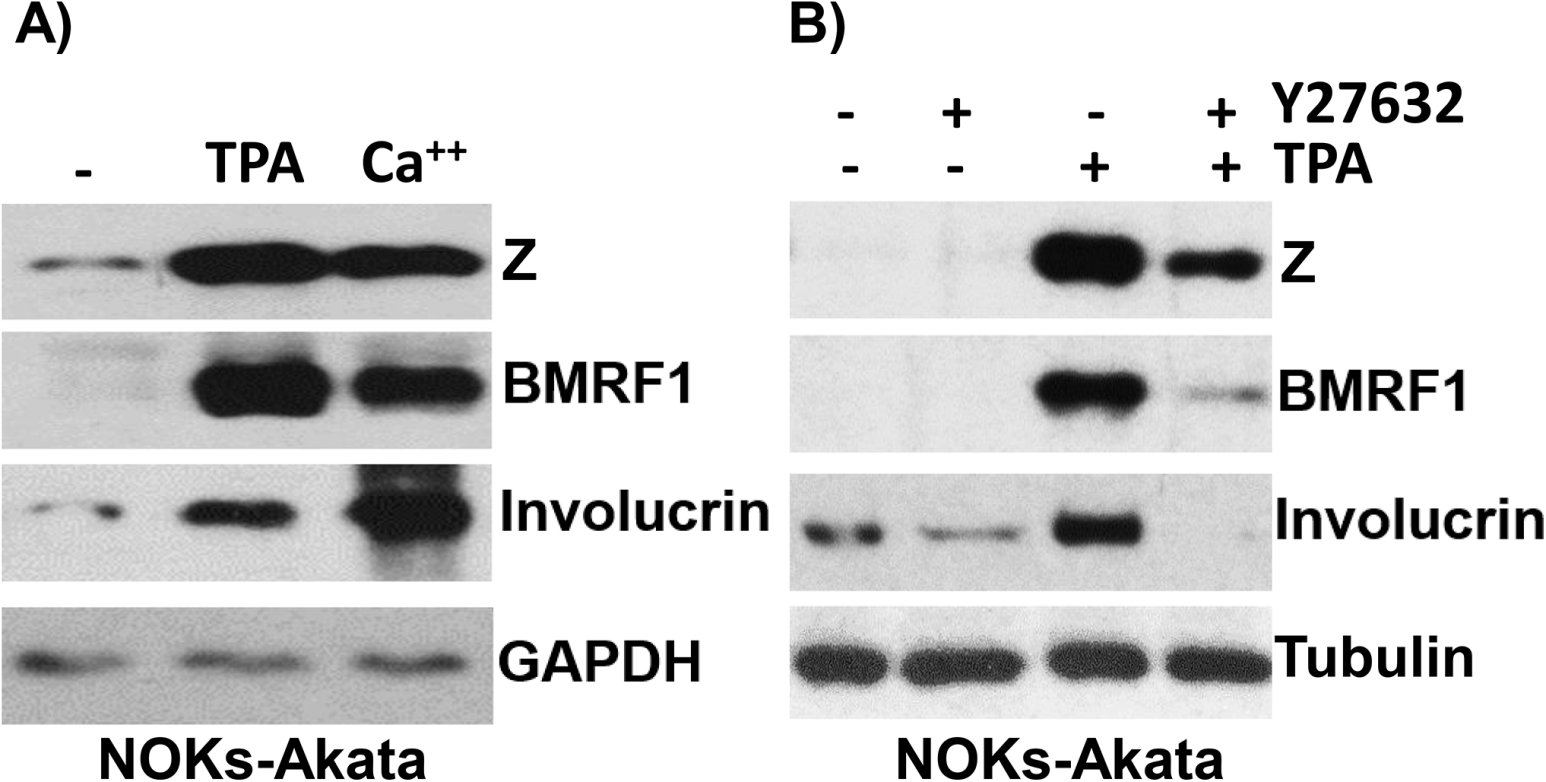
Treatment of NOKs-Akata cells with the differentiating agents, TPA and calcium chloride/serum, results in lytic EBV reactivation. A) NOKs-Akata cells were treated with either TPA (20 ng/mL in K-SFM) or calcium chloride plus serum (1.2 mM CaCl in RPMI + 10% FBS) for 48 hours. Immunoblot analysis was performed to compare the levels of lytic viral proteins, Z and BMRF1, a differentiation-dependent cellular protein, involucrin, and the cellular protein, GAPDH (a loading control). B) NOKs-Akata cells were treated with TPA in the presence or absence of a ROCK inhibitor (Y27632) (10 μM) for 48 hours and immunoblot analysis was performed to compare the levels of Z, BMRF1 and involucrin. The cellular protein tubulin was used as a loading control.

We next determined whether other treatments that have been reported to induce epithelial cell differentiation *in vitro*, such as phorbol ester (TPA), and calcium chloride/fetal bovine serum (FBS), can differentiate NOKs-Akata cells, and if they have any effect on lytic EBV reactivation. Treatment with either TPA or calcium chloride (given simultaneously with 10% FBS in RPMI media) promoted differentiation of NOKs-Akata cells, as indicated by increased expression of the differentiation-dependent cellular protein, involucrin (Fig 3A). Both treatments also led to lytic EBV reactivation, as indicated by increased expression of the lytic viral proteins, Z and BMRF1 (Fig 3A). To determine if TPA-mediated lytic reactivation of NOKs-Akata cells is at least partially differentiation-dependent, cells were treated with TPA in the presence or absence of a Rho-associated, coiled-coil-containing protein kinase (ROCK) inhibitor (Y27632) that has been reported to inhibit epithelial cell differentiation [43]. The ROCK inhibitor decreased both the TPA-induced differentiation marker (involucrin) and TPA-mediated lytic EBV reactivation (Fig 3B), suggesting that the TPA effect on EBV gene expression in NOKs-Akata cells is at least partially differentiation-dependent.

### The differentiation-dependent cellular transcription factor, KLF4, activates both the Rp and Zp IE EBV promoters in reporter gene assays, and induces lytic EBV reactivation in latently infected epithelial cell lines

Since lytic EBV reactivation in NOKs-Akata cells primarily depends upon activation of the viral Rp IE promoter [36], we next examined whether this promoter can be activated by the Kruppel-like factor 4 (KLF4) cellular transcription factor. KLF4 is selectively expressed in the upper spinous and granular layers of skin epithelial cells and is required for terminal epithelial cell differentiation in skin [40,41,44]. Although our lab previously showed that the cellular Sp1 transcription factor can bind to and activate the Rp [45], the effect of KLF4 (a member of the zinc-finger family of proteins that binds to Sp1-like sites) on Rp activity has not been reported. Of note, KLF4 was recently reported to activate the immediate-early EBV Z promoter in reporter gene assays via a motif that also binds Sp1 [46], although its ability to induce lytic reactivation in EBV-infected B cells or epithelial cells has not been examined.

**Fig 4:**
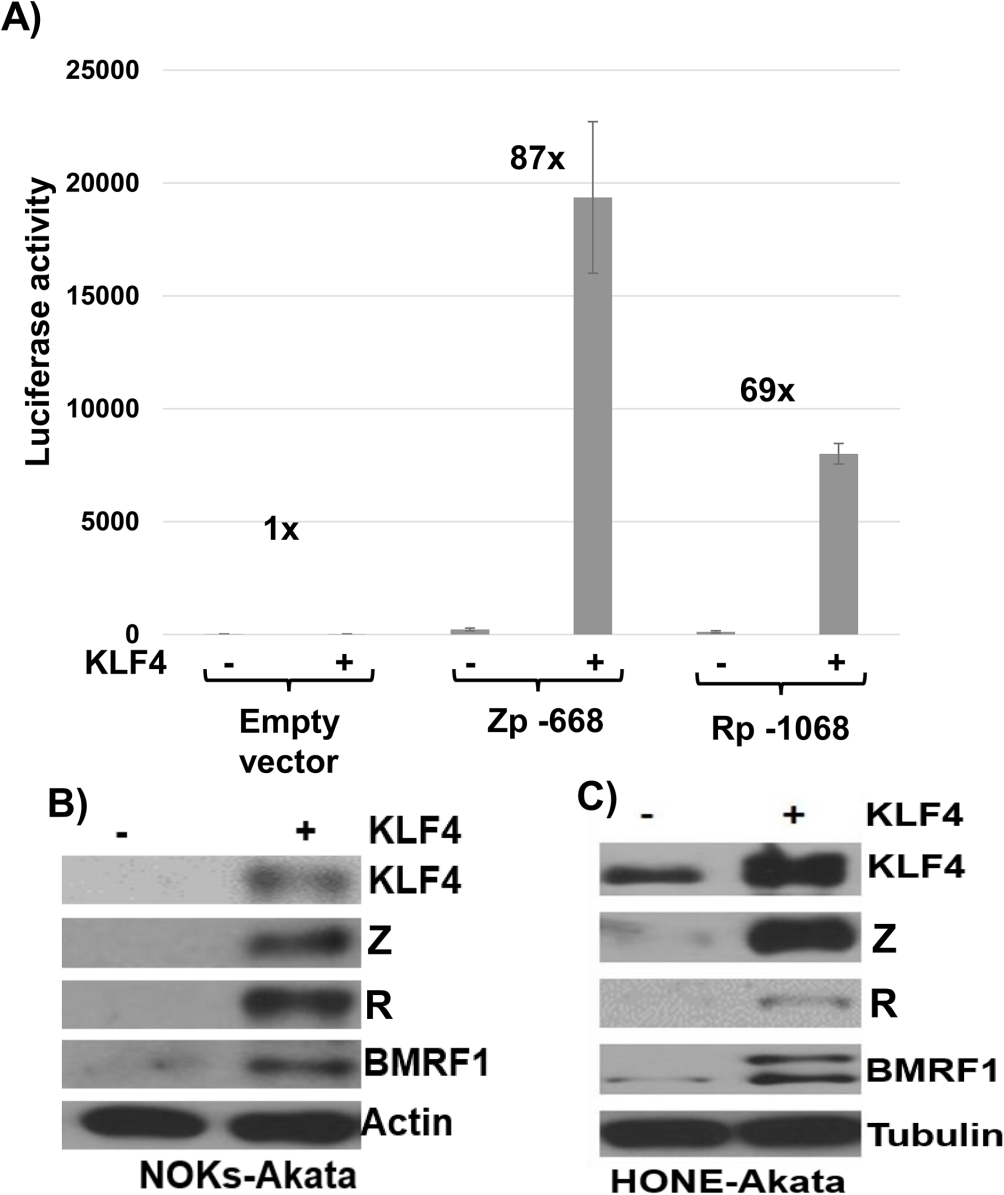
KLF4 activates both the Zp and Rp IE EBV promoters in reporter gene assays and induces lytic EBV reactivation when over-expressed in latently infected epithelial cells. A) Reporter gene constructs containing either the BZLF1 promoter (Zp-668), the BRLF1 promoter (Rp-1068), or no promoter sequences upstream of the luciferase gene were co-transfected into EBV-negative NOKs cells with either control vector or a KLF4 expression vector as indicated, and luciferase assays was performed 2 days after transfection. Total luciferase activity for each of the conditions from a representative experiment is shown (average +/- the standard deviation of results from three replicates), as well as the fold-increase in activity induced by KLF4. Similar results were obtained in three separate experiments. B) NOKs-Akata cells and C) HONE-Akata cells were transfected with either control vector or a KLF4 expression vector and immunoblot analysis was performed to compare the levels of transfected KLF4 and lytic viral proteins Z, R and BMRF1. Actin or tubulin served as a loading control.

As shown in Fig 4A, co-transfection of Rp- and Zp-driven luciferase vectors with a KLF4 expression vector greatly increased the activity of both EBV IE promoters in EBV-negative NOKs cells. Given the ability of KLF4 to activate both of the EBV IE promoters, we next asked if KLF4 over-expression is sufficient to induce lytic viral reactivation in latently infected NOKs-Akata cells (previously shown to be reactivated by R but not Z expression), or HONE-Akata cells (an epithelial carcinoma cell line, super-infected with the Akata strain of EBV, that can be reactivated by either Z or R expression) [36]. As shown in Fig 4B and 4C, overexpression of KLF4 was sufficient to activate expression of the EBV immediate-early proteins, Z and R, as well as the early lytic protein, BMRF1, in both epithelial cell lines. These results indicate that KLF4 is sufficient to induce lytic EBV reactivation in an epithelial cell environment.

### KLF4 is required for TPA and sodium butyrate mediated lytic EBV reactivation in epithelial cells

**Fig 5:**
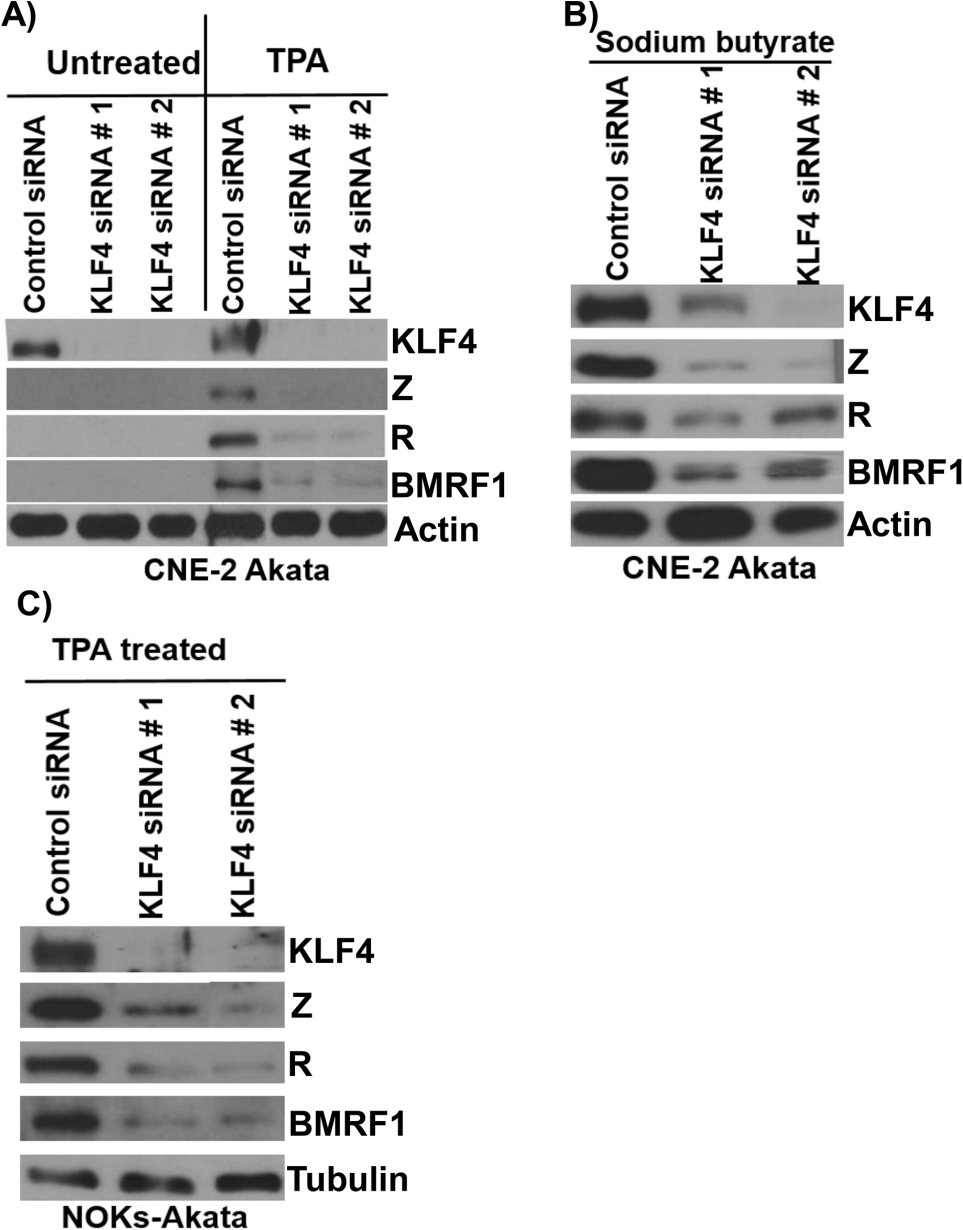
Endogenous KLF4 is required for TPA and sodium butyrate mediated lytic EBV reactivation in epithelial cell lines. EBV-positive CNE-2 Akata cells were transfected with either control siRNA or two different KLF4 siRNAs, and then treated with TPA (20 ng/uL) (A) or sodium butyrate (3mM) (B) for 48 hours. Immunoblot analysis was performed to detect expression of endogenous KLF4, and lytic viral proteins Z, R and BMRF1. Actin served as a loading control. C) NOKs-Akata cells, transfected with either control siRNA or KLF4 siRNAs, were treated with TPA (20 ng/uL) for 48 hours and immunoblot analysis was performed to compare the levels of endogenous KLF4 and lytic viral proteins Z, R and BMRF1. Tubulin served as a loading control.

Although expression of KLF4 is differentiation-dependent in normal epithelia, KLF4 is also commonly overexpressed in squamous cell carcinomas where it can act as an oncogene [47]. To determine whether endogenous KLF4 expression plays an important role in mediating lytic EBV reactivation in EBV-infected epithelial cell lines, KLF4 was knocked down using siRNA in two different EBV-positive epithelial cell lines, followed by treatment with TPA or sodium butyrate to induce lytic viral reactivation. As shown in Fig 5, CNE-2-Akata cells have detectable expression of KLF4, and knockdown of endogenous KLF4 inhibited the ability of both TPA and sodium butyrate treatment to induce lytic viral reactivation in CNE-2-Akata cells (Fig 5A and 5B). KLF4 knockdown also decreased the ability of TPA to induce lytic reactivation in NOKs-Akata cells (Fig 5C). Thus, constitutive KLF4 expression is required for efficient TPA-and sodium butyrate-mediated lytic EBV reactivation in EBV-infected epithelial cell lines.

### KLF4 activation of the Rp immediate-early promoter requires two consensus KLF4 binding motifs

**Fig 6:**
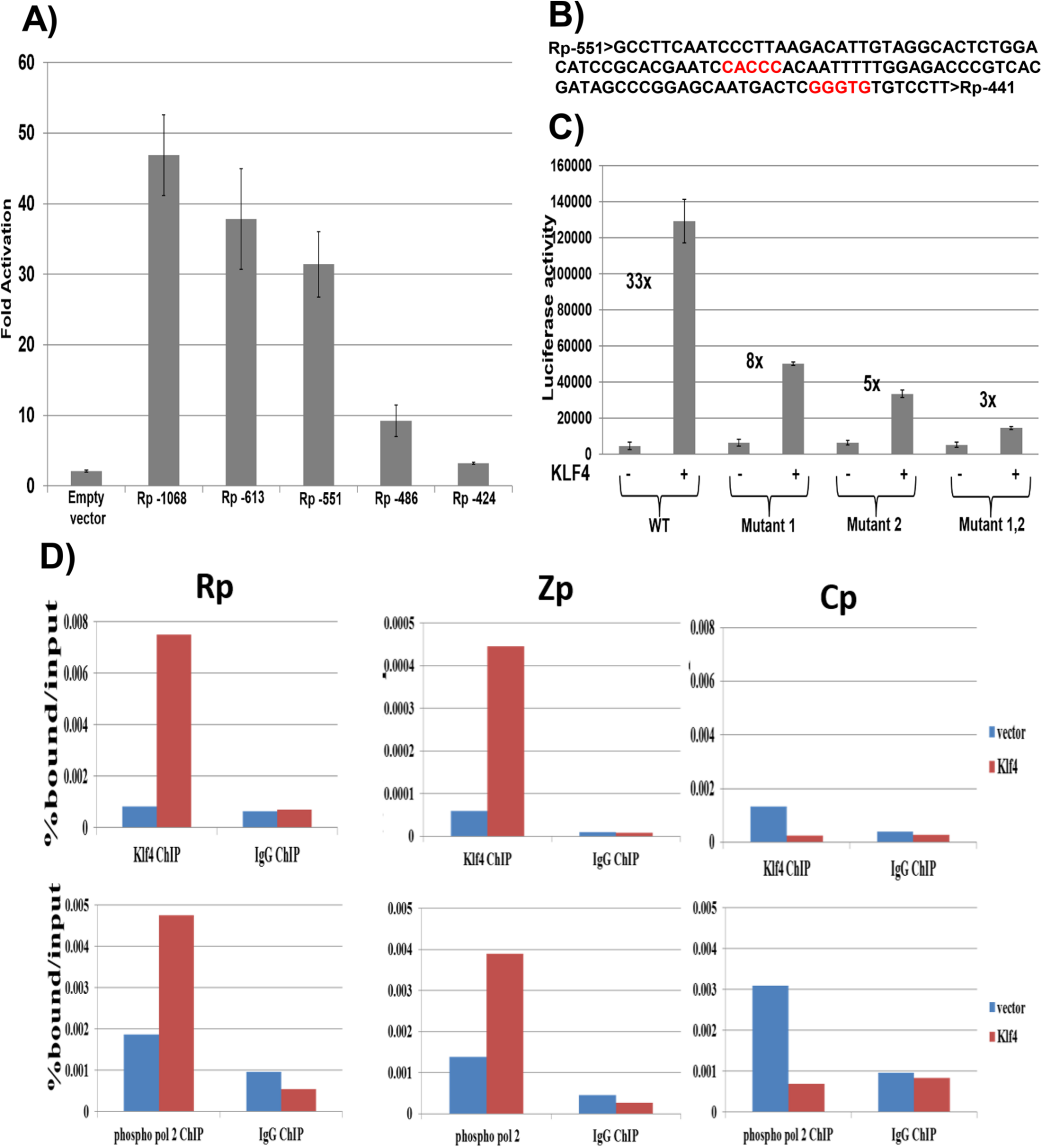
KLF4 binds to both the Zp and Rp IE EBV promoters, and enhances their association with activated RNA polymerase II. A) Various 5’ Rp deletion luciferase constructs were co-transfected into EBV-negative NOKs cells with either control vector or a KLF4 expression vector, and luciferase assay was performed 2 days after transfection. The KLF4-induced fold-change in luciferase activity for each construct is shown relative to the activity of the promoter in presence of control vector (set to 1). Values shown are the average +/- the standard deviation of results from two replicates. B) The EBV Rp sequence located between -551 and -441 relative to the transcriptional start site is shown. KLF4 consensus binding sites are highlighted in red. C) The Rp -551 construct (with or without KLF4 consensus site mutants) were co-transfected into EBV negative NOKs cells with control vector or KLF4 expression vector. Luciferase activity for each of the conditions is shown. Values are given as average +/- the standard deviations of results from two replicates. KLF4 mutant 1 alters the CACCC motif and mutant 2 alters the GGGTG motif. D) HONE-Akata cells were transfected with either control vector or a KLF4 expression vector, and ChIP assay was performed 48 hours after transfection. Cross-linked DNA-protein complexes were immunoprecipitated using anti-KLF4 antibody (top panel), or anti-phospho-RNA polymerase II antibody (bottom panel) and control IgG antibody in each case. Quantitative PCR was performed to quantitate the amount of DNA pulled down for the IE Rp (left panel), Zp (middle panel) and negative control Cp (right panel) EBV promoters.

To determine the mechanism by which KLF4 activates the BRLF1 promoter, we compared the ability of KLF4 to activate a series of 5’ Rp deletion mutants in reporter gene assays. KLF4 activation of Rp was significantly decreased when promoter sequences between -551 and -486 were deleted (Fig 6A). Site-directed mutagenesis was then performed to mutate two different consensus KLF4 motifs (located between -500 and -496, and between -452 and -448) (Fig 6B) alone, or in combination, in the Rp -551 luciferase construct. Mutation of either site alone partially decreased KLF4 activation of the promoter, and mutation of both sites simultaneously resulted in a more dramatic decrease (Fig 6C). This result suggests that both KLF4 consensus binding sites contribute to KLF4 activation of the BRLF1 promoter.

### KLF4 binds to the Zp and Rp EBV IE promoters *in vivo*, and enhances their association with activated RNA polymerase II

To examine whether KLF4 associates directly with the EBV Rp and/or Zp promoters *in vivo*, ChIP assays were performed in latently infected HONE-Akata cells transfected with either control vector or a KLF4 expression vector. These studies confirmed that KLF4 binds to both the Rp and Zp promoters of the endogenous viral genome in EBV-infected HONE cells (Fig 6D), but not to the EBV EBNA promoter, Cp. ChIP for RNA polymerase II (pol2) also revealed that KLF4 increased Rp and Zp occupancy with active pol2 (Fig 6D), consistent with its ability to activate transcription from both viral IE promoters. Similar results were obtained in NOKs-Akata cells (S3 Fig).

### KLF4 synergizes with BLIMP1 to activate both Rp and Zp

**Fig 7:**
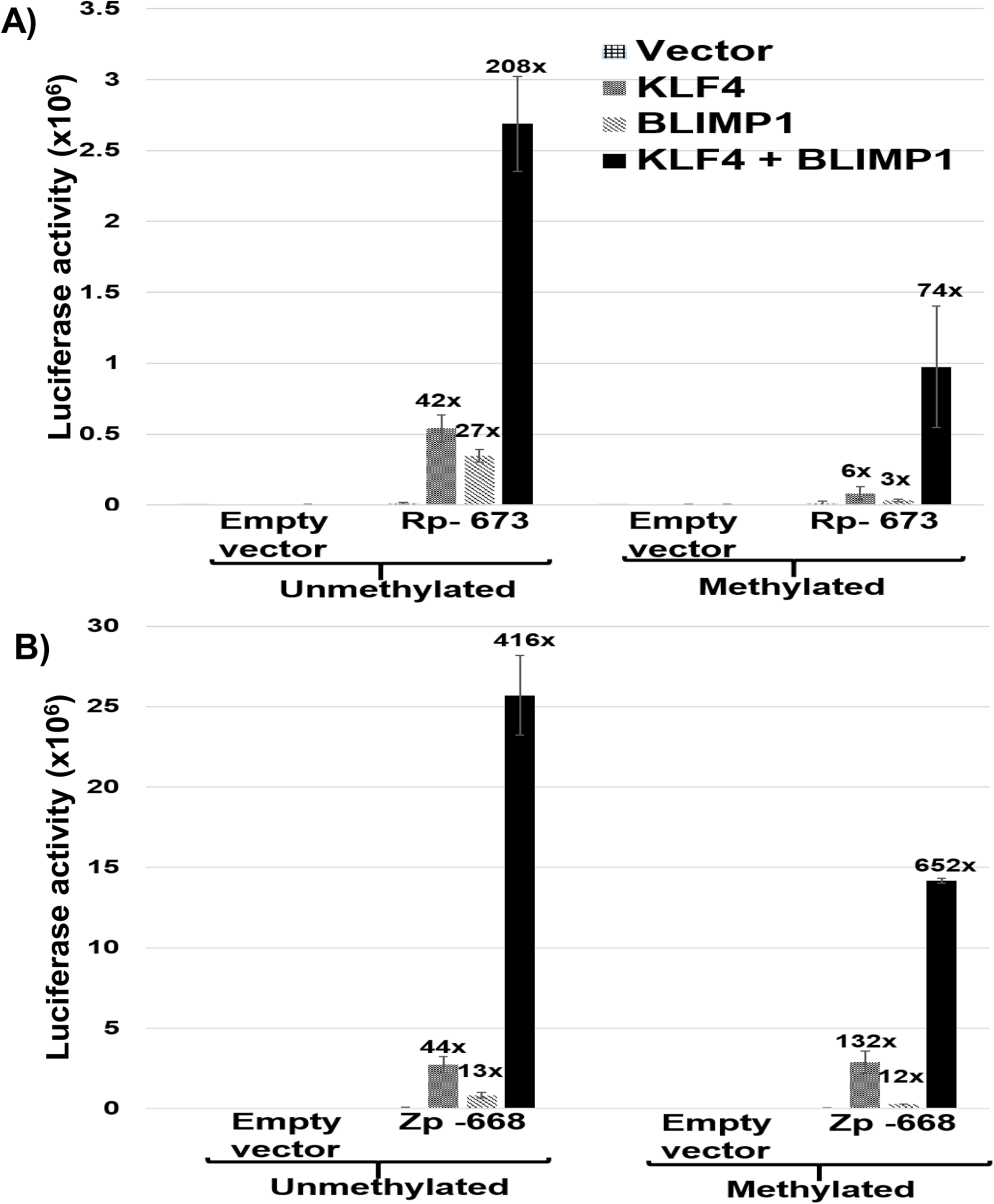
KLF4 synergizes with BLIMP1 to activate both Rp and Zp, irrespective of the promoter methylation state. A) Mock treated or methylated promoterless (empty) and Rp-673 pCpGL luciferase constructs were co-transfected into EBV-negative NOKs cells with either control vector, KLF4 vector alone, BLIMP1 vector alone, or the combination of KLF4 and BLIMP1 vectors. Luciferase activity for each of the conditions is shown; values are given as average +/- the standard deviations of results from two replicates. B) Mock treated or methylated promoterless vector and Zp-668 pCpGL luciferase constructs were co-transfected into EBV-negative NOKs cells with control vector, KLF4 vector, BLIMP1 vector or KLF4 and BLIMP1. Luciferase activity for each of the conditions is shown; values are given as average +/- the standard deviations of results from two replicates.

The cellular transcription factor, BLIMP1, activates both Rp [39] and Zp [48] in reporter gene assays, and, like KLF4, is induced during epithelial cell differentiation. To determine if the combination of BLIMP1 and KLF4 activates the Rp and/or Zp promoters more strongly than either BLIMP1 or KLF4 alone, we compared the effects of each transcription factor alone, or in combination, on Zp and Rp activity in EBV-negative NOKs cells. Since the ability of KLF4 to activate some promoters is affected by promoter methylation [49,50], we also examined whether DNA methylation of the Rp or Zp promoters alters the KLF4 and/or BLIMP1 effect. As shown in Fig 7A and 7B, the combination of KLF4 and BLIMP1 together produced highly synergistic activation of both the Rp and Zp promoters, regardless of promoter methylation status. Of note, the ability of KLF4 and BLIMP1 alone to activate the Rp -673 promoter construct was significantly reduced when the promoter was methylated, while activation mediated by the BLIMP1/KLF4 combination was less affected. Thus, the availability of both KLF4 and BLIMP1 may be particularly important for activating the Rp in situations where it is highly methylated (as occurs in EBV-positive NPC [51]). Conversely, methylation of Rp and loss of either KLF4 and/or BLIMP1 expression might be one of the mechanisms by which EBV achieves latency in these tumor cells.

### KLF4 synergizes with BLIMP1 to induce lytic EBV reactivation and DNA replication in latently infected epithelial cells

**Fig 8:**
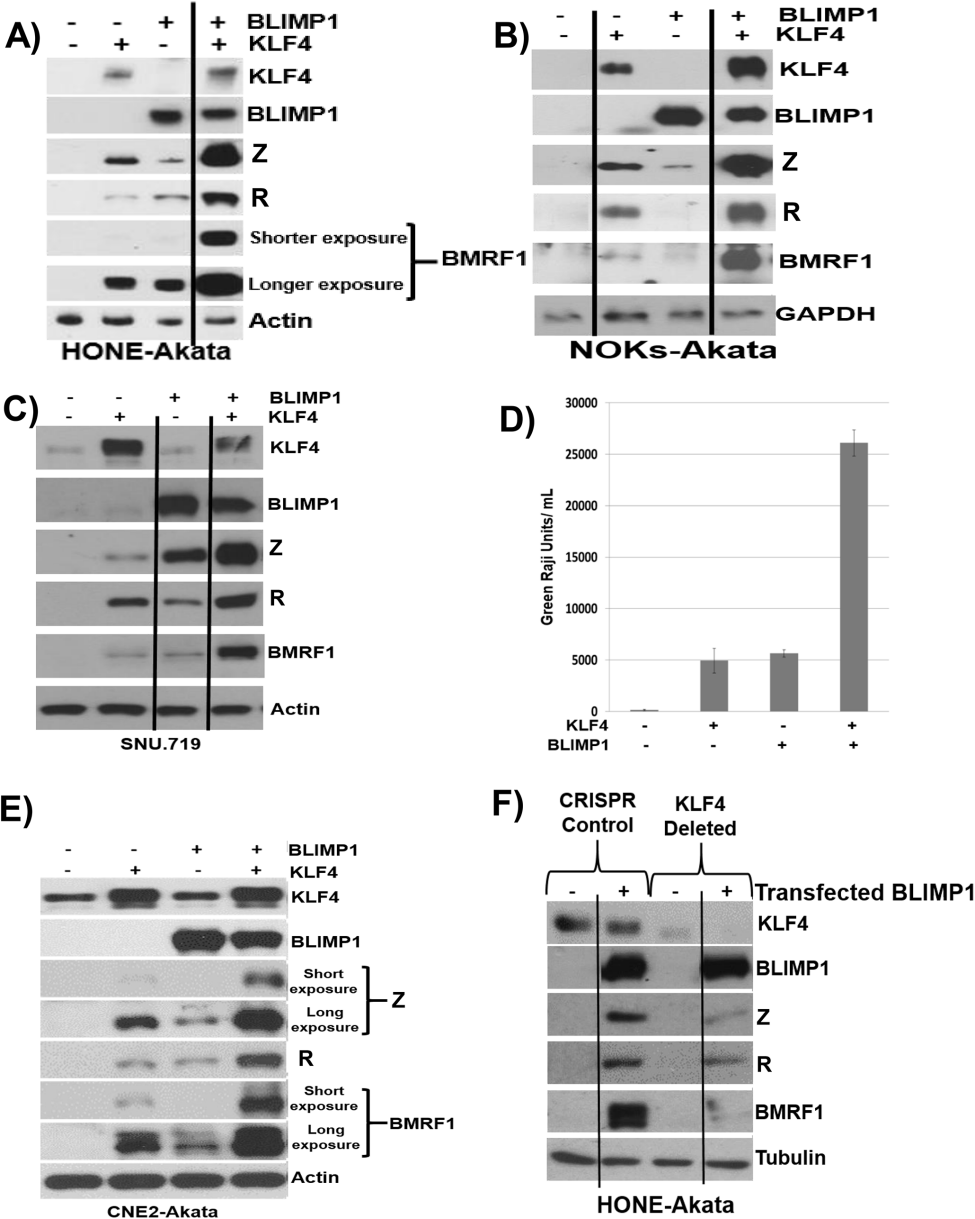
KLF4 synergizes with BLIMP1 to induce lytic EBV reactivation and replication in latently infected epithelial cells. Control vector or KLF4 and BLIMP1 expression vectors (either alone or in combination) were transfected into A) HONE-Akata cells, B) NOKs-Akata cells, or C) SNU.719 gastric carcinoma cells and immunoblot analysis was performed to compare the levels of transfected KLF4 and BLIMP1, and induction of EBV lytic viral proteins Z, R and BMRF1. Actin or GAPDH served as a loading control. D) An infectious viral titer assay was performed, as described in materials and methods, using the supernatant of CNE2-Akata cells transfected with vector control, KLF4, BLIMP1, and KLF4 and BLIMP1, five days post transfection. E) Immunoblot analysis for the samples shown in Fig 8D. F) HONE-Akata cells in which KLF4 was knocked-out using CRISPR-Cas9 technology or control HONE-Akata cells were transfected with either control vector or a BLIMP1 expression vector and immunoblot analysis was performed to compare the levels of endogenous KLF4, transfected BLIMP1, and lytic viral proteins Z, R and BMRF1. Tubulin served as a loading control.

To determine whether KLF4 also synergizes with BLIMP1 to induce lytic reactivation in latently infected EBV-positive epithelial cell lines, we transfected KLF4 and BLIMP1 expression vectors (alone or in combination) into HONE-Akata, NOKs-Akata or SNU.719 cells (an EBV-positive gastric carcinoma line). While BLIMP1 and KLF4 alone both induced detectable expression of the two IE proteins (Z and R), as well as the early protein, BMRF1, in each cell line, the combination of KLF4 and BLIMP1 together produced dramatically more lytic EBV protein expression than either KLF4 or BLIMP1 alone in each line (Fig 8A–8C). KLF4 also synergized with BLIMP1 to induce the expression of late viral capsid protein, p18, in EBV-infected HONE, NOKs and SNU.719 cells (S4 Fig). Likewise, the combination of KLF4 and BLIMP1 synergistically increased the release of infectious virion particles from CNE-2 Akata cells (Fig 8D and 8E), and the amount of intracellular EBV DNA in HONE-Akata cells (S4 Fig). Of note, neither KLF4 nor BLIMP1 induced expression of the other protein in epithelial cells.

Since we observed constitutive KLF4 (but not BLIMP1) expression in a number of EBV-infected epithelial cell lines (Fig 5), we also determined whether endogenous KLF4 expression is required for BLIMP1-mediated lytic EBV reactivation. The ability of transfected BLIMP1 to induce expression of EBV lytic proteins was compared in HONE-Akata cells where endogenous KLF4 expression was knocked out using CRISPR-Cas9 technology, versus HONE-Akata cells infected with a non-targeting control CRISPR-Cas9 vector. Disruption of the KLF4 gene significantly reduced the ability of BLIMP1 to induce lytic EBV gene expression (Fig 8F). These results confirm that KLF4 and BLIMP1 strongly collaborate to induce lytic EBV reactivation, and suggest that differentiation-associated lytic reactivation in normal epithelial cells is likely to be at least partially mediated through KLF4 and BLIMP1. We were unable to create a derivative of NOKs-Akata cell line in which KLF4 was stably knocked down or knocked out using either shRNA or CRISPR-Cas9 technology, respectively, suggesting that KLF4 serves as an essential survival factor for this cell line.

### KLF4 and BLIMP1 expression is differentiation-dependent in normal tongue epithelium, and OHL cells express KLF4 and BLIMP1

**Fig 9:**
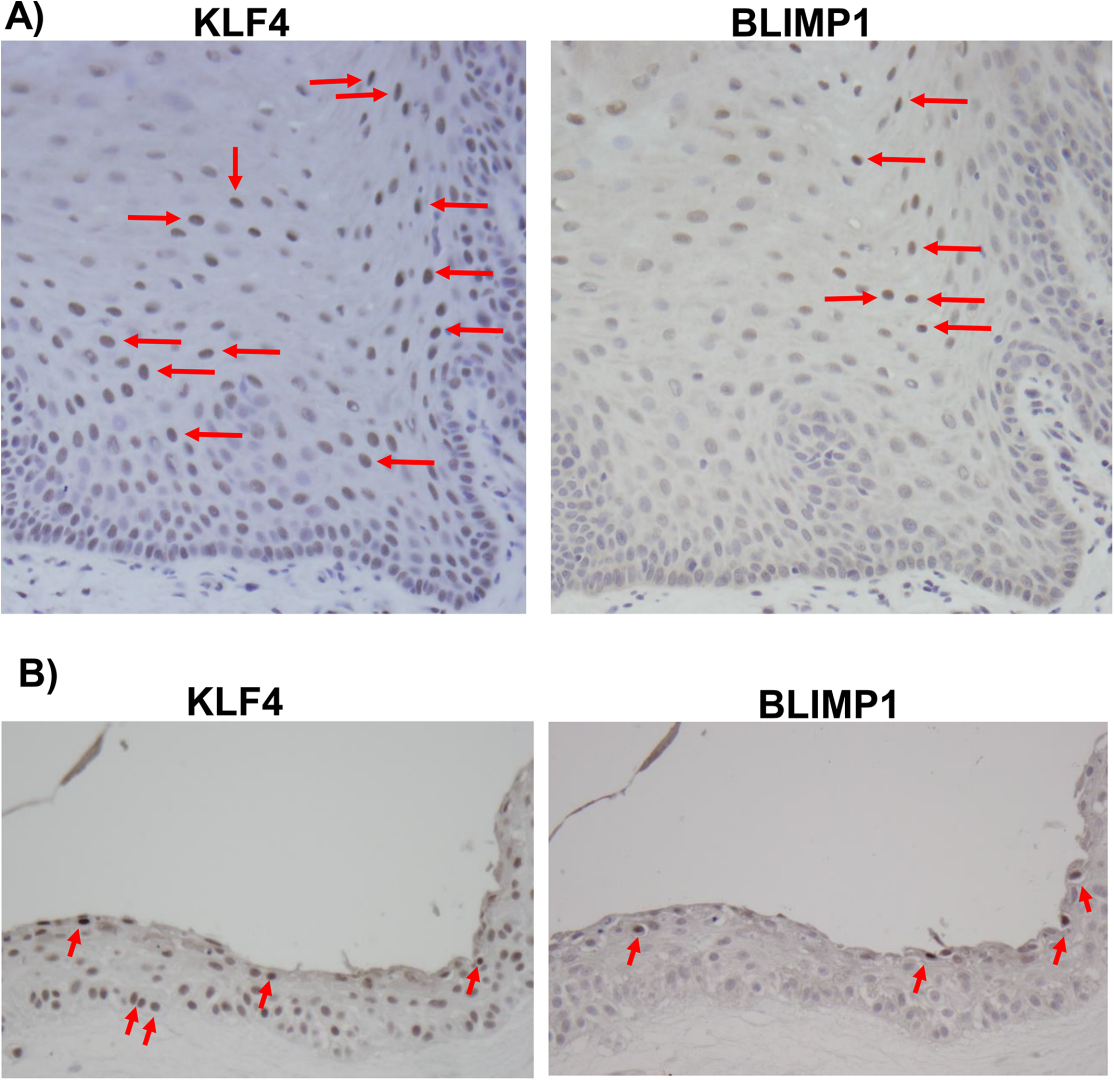
KLF4 and BLIMP1 expression is differentiation-dependent in normal tongue tissue. Immunohistochemistry analysis was performed on paraffin-embedded formalin-fixed normal tongue tissue (A), or rafted NOKs-Akata cells (B), using antibodies directed against KLF4 and BLIMP1 as indicated. Examples of positively-staining cells are indicated by red arrows (Images:40x).

Although previous studies have shown that expression of KLF4 is differentiation-dependent in normal skin [41,44], to our knowledge the effect of differentiation on the expression of KLF4 in human oral mucosal epithelium has not been examined. To determine whether KLF4 and BLIMP1 expression is regulated by differentiation in normal human tongue tissue, we performed immunohistochemistry analysis using antibodies directed against these two cellular proteins. Expression of both KLF4 and BLIMP1 was highest in cells within the suprabasal compartment (Fig 9A). Similar results were obtained in normal human tonsil epithelium (S5 Fig). In NOKs-Akata cells grown in raft cultures, BLIMP1 expression was differentiation dependent. However, KLF4 was expressed in a portion of undifferentiated basal epithelial cells as well as in the more differentiated cells (Fig 9B), consistent with our finding that KLF4 is required for the long-term survival of this cell line.

**Fig 10:**
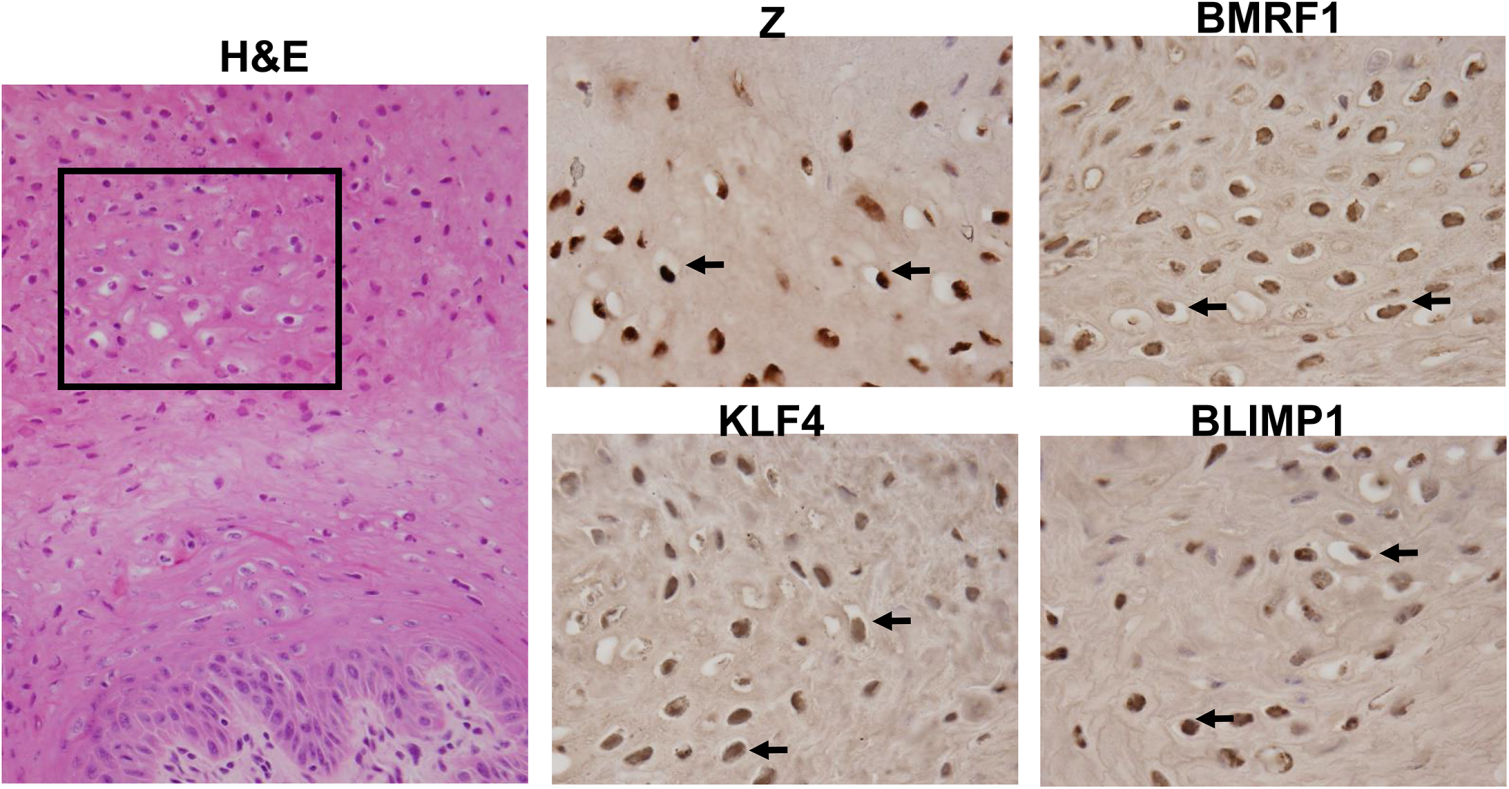
OHL cells express both KLF4 and BLIMP1. H&E analysis (Image: 40X), and immunohistochemistry analysis was performed on a paraffin-embedded, formalin-fixed biopsy of an oral hairy leukoplakia (OHL) lesion using antibodies directed against Z, BMRF1, KLF4 and BLIMP1 as indicated (Images: 100x of region boxed in H & E stain). Examples of OHL cells, positively staining for each of these proteins, are indicated by black arrows.

To confirm that KLF4 and BLIMP1 are also both expressed in the highly lytic OHL lesions that can occur within differentiated tongue epithelium of immunosuppressed patients, we examined KLF4 and BLIMP1 expression in a patient-derived OHL lesion. We confirmed that cells with the morphology typical of OHL cells (“ballooning” cells with a large amount of cytoplasm- see arrows) not only expressed the lytic viral proteins, Z and BMRF1, but also the cellular proteins, KLF4 and BLIMP1 (Fig 10). Collectively, these results suggest that the expression of KLF4 and BLIMP1 correlates with lytic EBV infection in the tongue.

### Lytic EBV infection occurs in differentiated oropharyngeal epithelial cells in humans, while undifferentiated cells may support a small level of latent infection

To examine further the effect of epithelial cell differentiation state on EBV gene expression in humans, and to explore the possibility that undifferentiated cells can support low-level latent EBV infection, epithelial cells were isolated using laser dissection from the undifferentiated (basal), partially differentiated (middle), or differentiated (superficial) layers of normal tonsil epithelium, or OHL lesions; only normal tonsils that had EBER+ B cells were examined (S6 Fig). Total RNA was purified, DNase treated, reverse transcribed, and amplified with a series of primers (Table 1) to detect both epithelial cell-specific, B cell-specific, and viral transcripts. As shown in Table 2, the cellular ∆Np63 transcript (expressed specifically in basal epithelial cells) was detected in basal, but not superficial, OHL lesions, confirming our ability to separate these layers accurately. Furthermore, the B-cell-specific CD20 transcript was not detected (using primers that can detect even one CD20-positive cell in 1000 cells), making it very unlikely that the isolated RNA was partially derived from infiltrating B cells. In the majority (3/4) of OHL tissue, lytic EBV transcripts (including BZLF1, BRLF1, and BcLF1) could not be detected in the undifferentiated epithelial cells, but were easily detected in differentiated cells. Interestingly, however, EBER transcripts were detected in the basal epithelial cells in the majority (3/4) of OHL biopsies (Table 2). Furthermore, EBER transcripts were also detected in undifferentiated basal cells in normal tonsil tissue, although lytic EBV transcripts were not detected (Table 3). In addition, low level EBER staining was detected in undifferentiated epithelium of tonsils that had a high number of EBER-positive B cells (S6 Fig). These results suggest that EBV, like HPV, may establish persistent latent infection in some basal epithelial cells, and confirm that lytic EBV reactivation is confined to more differentiated epithelial cell layers.

**Table 1:** Primers used for RT-qPCR studies.

Transcript	Primer	Sequence (5’-3’)
EBER1	5’ primer	CGTCCCGGGTACAAGTCC
EBER1	3’ primer	AAGACGGCAGAAAGCAGAGTCT
BcLF1	5’ primer	CTCGTTGACCATGTTGTA
BcLF1	3’ primer	CTGGGTGACATCATGTAC
BZLF1	5’ primer	CTTGGCCCGGCATTTTCT
BZLF1	3’ primer	ACGACGCACACGGAAACC
BRLF1	5’ primer	TGGCTTGGAAGACTTTCTGAGGCT
BRLF1	3’ primer	AATCTCCACACTCCCGGCTGTAAA
ΔNP63	5’ primer	GAAGAAAGGACAGCAGCATTGAT
ΔNP63	3’ primer	GGGACTGGTGGACGAGGAG
CD20	5’ primer	CAGTGTGCTTGAGAAACAAAC
CD20	3’ primer	CAGGATCTGAGTCTCCAAGG
GAPDH	5’ primer	GGGAAGCTTGTCATCAATGGA
GAPDH	3’ primer	CGCCCCACTTGATTTTGG
Cyclophilin A	5’ primer	CTTGGGCCGCGTCTCC
Cyclophilin A	3’ primer	GCAGGAACCCTTATAACCAAATCC

**Table 2:** EBV transcript expression OHL lesions.

L samples	GAPDH (Ct)	CD20 (Ct)	EBER1 (Ct)	BZLF1 (Ct)	BRLF1 (Ct)	BcLF1 (Ct)	ΔNP63 (Ct)
OHL-1							
Basal	30.60±0.30	-	-	-	-	-	37.26±1.22
Middle	32.06±0.03	-	31.71±0.18	37.82±0.99	34.11±0.37	35.24±0.54	-
Superficial	35.13±1.17	-	34.88±1.05	-	35.08±0.63	-	-
OHL-2							
Basal	29.07±0.88	-	34.95±0.83	-	-	-	37.62±0.06
Middle	29.68±0.03	-	29.45±0.35	ND	ND	35.85±2.13	ND
Superficial	37.09±2.11	-	32.37±0.60	-	35.62±1.58	38.27±0.51	-
OHL-3							
Basal	25.44±0.52	-	32.49±1.32	-	33.51±1.60	34.99±1.05	30.22±1.09
Middle	28.23±0.10	-	24.04±0.04	36.49±0.16	26.51±0.19	28.10±0.12	32.21±0.36
Superficial	34.23±1.39	-	25.14±0.13	-	27.18±0.06	28.56±0.07	-
OHL-4							
Basal	29.71±0.13	-	34.49±1.55	-	-	-	39.24±0.17
Middle	36.49±3.09	-	31.47±0.57	34.95±0.09	33.96±0.54	36.07±0.95	-
Superficial	-	-	34.31±1.00	-	-	-	-

**Table 3:** EBV transcripts in normal tonsil tissue.

Normal Tonsil	CycA (Ct)	CD20 (Ct)	EBER1 (Ct)	BRLF1 (Ct)	BcLF1 (Ct)	ΔNP63 (Ct)
1						
Basal	31.87±0.08	-	37.11±0.11	-	-	35.64±0.04
Middle	30.35±0.10	-	-	-	-	35.08±0.21
Superficial	-	-	-	-	-	-
2						
Basal	27.08±0.03	-	38.66±0.34	-	-	30.68±0.38
Middle	30.46±0.17	-	-	-	-	34.90±0.66
Superficial	31.24±0.11	-	-	-	-	37.07±0.02
3						
Basal	21.89±0.07	-	39.36±0.07	-	-	24.36±0.12
Middle	24.80±0.49	-	-	-	-	28.94±0.38
Superficial	27.52±0.39	-	-	-	-	-
4						
Basal	23.72±0.09	-	38.77±0.08	-	-	25.98±0.09
Middle	23.71±0.02	-	-	-	-	28.36±0.14
Superficial	37.14±1.59	-	-	-	-	-
5						
Basal	28.25±0.03	-	32.43±0.44	-	-	33.74±0.40
Middle	31.75±0.18	-	-	-	-	36.54±0.11

### KLF4 protein is not detectably expressed in B cells

**Fig 11:**
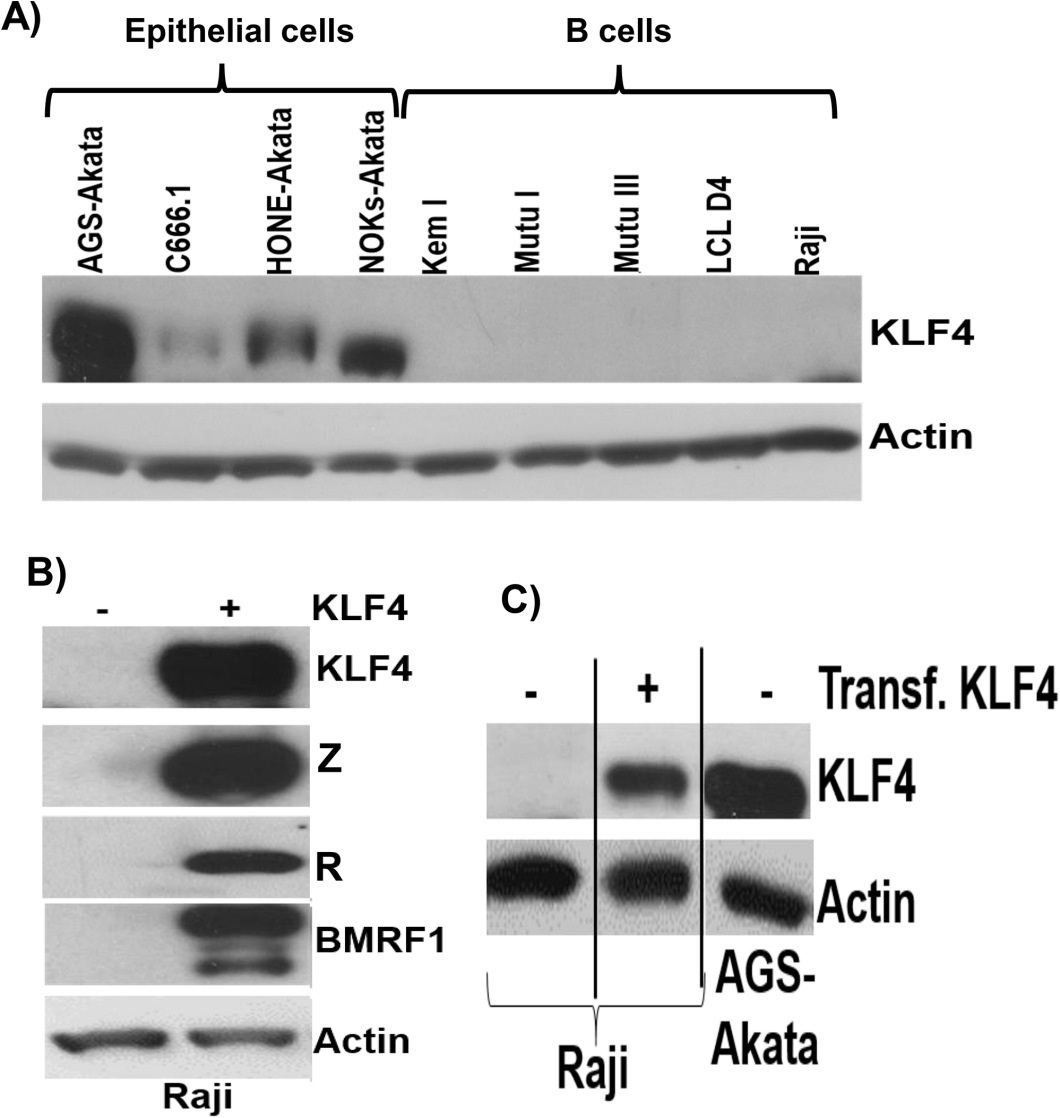
KLF4 is not expressed in B cells but can reactivate lytic EBV gene expression in this cell type. A) Immunoblot analysis was performed to compare the levels of endogenous KLF4 expression in a series of EBV-infected epithelial cell lines and EBV-infected B cell lines. Actin served as a loading control. B) Raji Burkitt lymphoma cells were transfected with either control vector or a KLF4 expression vector and immunoblot analysis was performed to compare the levels of transfected KLF4 and lytic viral proteins Z, R and BMRF1. Actin served as a loading control. C) The levels of transfected KLF4 into Raji cells (from 11B) was compared to the endogenous levels of KLF4 in AGS-Akata cells.

Very little is known regarding KLF4 protein expression in B cells, in which EBV normally enters the latent form of infection. To further examine the potential role of KLF4 in regulating EBV gene expression in B cells, we performed immunoblot analysis to compare KLF4 levels in a variety of EBV-infected epithelial and B cell lines. Interestingly, we found that the cell line with the highest level of KLF4 expression (AGS-Akata) is an EBV-superinfected gastric carcinoma line that we previously reported supports unusually high level lytic EBV protein expression [52] (Fig 11A). Consistent with a previous report showing that KLF4 expression is decreased in NPC tumor specimens [53,54], we found that KLF4 expression in C666.1 cells (the only authentic EBV+ NPC tumor cell line in this panel) is relatively low compared to that in the EBV-superinfected epithelial cell lines (NOKs and HONE). Furthermore, KLF4 was not detected in either EBV-positive Burkitt lines or EBV-transformed lymphoblastoid cells. Consistent with the lack of KLF4 protein expression in Burkitt cell lines, Human Protein Atlas studies also recently reported no detectable KLF4 protein expression in the lymphoid tissue of normal human tonsil or spleen [55].

To determine if KLF4 expression can be induced in Burkitt lymphoma cells treated with agents that activate the lytic form of EBV infection, Burkitt cells were treated with a variety of different lytic-inducing agents, including anti-IgG (which induces B-cell receptor activation), 5-aza-2’-deoxycytidine (a demethylating agent) and TGF-β. These treatments did not restore KLF4 expression in Burkitt cells, although these agents induced lytic EBV reactivation as expected (S7 Fig). Nevertheless, overexpression of KLF4 in both the Raji and Jijoye latently infected, EBV-positive, Burkitt lymphoma cell lines was sufficient to induce the expression of the EBV immediate-early proteins, Z and R, as well as the early lytic protein, BMRF1 (Figs 11B and 11C and S8). Thus, KLF4 can induce lytic EBV reactivation even in a B cell-environment and, hence, the lack of KLF4 expression in this cell type may promote viral latency.

## Discussion

EBV infection of oropharyngeal epithelial cells is associated with both malignant and non-malignant human diseases, but the viral and cellular factors that regulate EBV gene expression in epithelial cells are poorly understood. Here, we show in both *in vitro* and *in vivo* studies that the induction of differentiation of epithelial cells promotes the lytic form of EBV infection, while undifferentiated basal epithelial cells support latent EBV infection. In addition, we demonstrate that the differentiation-dependent cellular transcription factors, KLF4 and BLIMP1, collaboratively link EBV lytic reactivation to epithelial cell differentiation. Furthermore, we find that the absence of KLF4 expression in B cells may help to promote viral latency in this cell type. Together, these studies suggest that KLF4 plays a key role in regulating EBV gene expression, particularly in epithelial cells.

KLF4, a zinc-finger protein that binds to Sp1-like sites, can act as either a positive or negative regulator of transcription when bound to promoters, and has highly divergent functions depending upon the cell type and context in which it is expressed [56–61]. KLF4 is perhaps best known for its ability to convert differentiated cells into IPS cells when delivered with three other cellular transcription factors, Oct4, Sox2 and c-Myc [62]. In this context, KLF4 has been reported to act as a “pioneer” transcription factor that can bind to DNA sites within heterochromatin, and subsequently convert the chromatin to a more open form that can then be bound by other transcription factors such as c-myc [63]. However, KLF4 also inhibits gene expression in some instances, by binding to co-repressor proteins such as HDACs [64].

The role of KLF4 in tumor formation is also very complex, as KLF4 can function as either a tumor suppressor, or tumor promoter, depending upon the cell type and other factors. KLF4 is thought to function as a tumor suppressor in certain epithelial cell tumors, and its expression is decreased in both EBV-positive NPC and in gastric carcinoma [54,65–67]. Consistent with its role as a tumor suppressor, KLF4 inhibits cell cycle progression in some, but not all, cell types [59,68]. In the case of normal epithelium, KLF4 is expressed in differentiated, but not undifferentiated, cells, and the knockout of the KLF4 gene in mice results in abnormal epithelial cell differentiation [40]. Furthermore, KLF4 binds to the involucrin gene promoter, and mediates differentiation-dependent expression of this gene, in human epithelium [44]. The results here suggest that KLF4 likewise helps to promote differentiation-dependent expression of the two EBV IE genes in oral epithelium.

However, KLF4 may also function as a tumor promoter in some types of epithelial cell carcinomas, including squamous cell carcinomas (SCC) [47]. Consistent with a survival role for KLF4 in certain epithelial cell tumors, KLF4 induces squamous epithelial cell dysplasia when expressed in basal keratinocytes in mice [69], transforms rat kidney epithelial cells *in vitro* [70], and is correlated with bad prognosis when over-expressed in human head and neck carcinomas [47,71]. The mechanism(s) by which KLF4 switches to a tumor activator in certain types of epithelial cell carcinoma are not totally understood, but may be related to the multiple different types of post-translational modifications that can occur on the KLF4 protein (including phosphorylation, acetylation and sumoylation) and alter its functions [72–77]. Similar to its potential role as an oncogene in certain SCCs, we found that KLF4 is required for long-term survival of NOKs cells and thus we were unable to knockdown KLF4 expression (except transiently) in this cell type.

In B cells, KLF4 has been reported to halt cell cycle progression and act as a tumor suppressor [78–80]. Even though KLF4 transcripts can be detected in normal B cells, recent immunohistochemistry staining studies reported by the Human Protein Atlas consortium indicated that KLF4 protein is undetectable within the lymphoid regions of normal tonsils, spleen and lymph nodes [55]. We were likewise unable to detect KLF4 protein expression in any of the EBV-infected B cell types that we examined by immunoblot analysis (Fig 11), including Burkitt lymphoma cell lines with various forms of EBV latency and EBV-transformed lymphoblastoid cell lines, and did not detect KLF4 expression (by IHC analysis) in the lymphoid areas of normal human tonsil and spleen. The absence of KLF4 expression in EBV-infected B cells may provide a mechanism to promote viral latency. In any event, our finding that KLF4 expression is sufficient to induce lytic EBV gene expression in Burkitt lymphoma cell lines indicates that KLF4 can promote lytic EBV gene expression outside the context of epithelial cell differentiation.

We also show here that KLF4 binds to the endogenous viral Rp IE promoter in EBV-infected HONE and NOKs cells, and that KLF4 binding sites in the promoter are required for efficient KLF4-mediated activation of the promoter in reporter gene assays. In addition, we show that KLF4 also binds to the Zp IE promoter *in vivo*. This result is consistent with the report of another group showing that KLF4 can activate Zp in a reporter gene assay through previously mapped Sp1 motifs [46]. Since Sp1 has been shown to bind to and activate the Zp and Rp promoters *in vitro* [45,46], and KLF4 and Sp1 bind to the same motif, it has not been clear what role, if any, KLF4 plays in mediating viral reactivation *in vivo*. Our results here suggest that KLF4 is critical for promoting efficient lytic reactivation during normal epithelial cell differentiation, but is less likely to play an important role in B cells, since B cells do not appear to express KLF4 protein within normal lymphoid tissue. Nevertheless, it remains possible that KLF4 can be activated in EBV-infected B cells under certain circumstances and contribute to lytic reactivation in specific contexts.

We also show here that the combination of the KLF4 and BLIMP1 transcription factors results in much more lytic EBV gene expression than either factor alone. This synergistic effect on EBV IE promoter activity may be most biologically relevant in differentiating epithelial cells such as normal tongue tissue, where we show that the two transcription factors are coordinately expressed. Since we found no evidence that BLIMP1 affects KLF4 expression, or vice versa, we postulate that the two transcription factors independently activate Zp and Rp transcription through different mechanisms, and that the combined effect of these two factors is synergistic. BLIMP1 has been previously shown to activate Zp in reporter gene assays [48], and we recently reported that BLIMP1 can also activate Rp in both EBV-negative epithelial cells and B cells [39]. BLIMP1 activation of Rp is at least partially mediated through Rp sequences centered at -660 relative to the transcriptional start site [39]. Although BLIMP1 is complexed to Rp *in vivo* in ChIP assays, it does not bind to this site directly *in vitro*, based upon EMSA results [39], and the exact mechanism(s) by which BLIMP1 activates either Rp or Zp has not been clearly defined. Since BLIMP1 binding to promoters usually results in inhibition of promoter activity [81–84], it is possible that BLIMP1 down-regulates a cellular factor(s) which in turn inhibits Zp and Rp activity. As BLIMP1 expression is increased by differentiation of both epithelial and plasma cells, it likely plays a role in the lytic reactivation of EBV in both cell types. Our finding that endogenous KLF4 expression in epithelial cells is required for efficient BLIMP1-mediated lytic reactivation in that cell type raises the interesting prospect that another member of the Kruppel-like cellular transcription factor family serves a similar role in differentiation- dependent lytic reactivation in plasma cells.

Finally, our studies examining the expression of latent (EBER) and lytic EBV transcripts in different layers of OHL lesions and normal tonsil epithelium confirm that lytic EBV transcripts are largely confined to differentiated epithelium in humans, although EBER transcripts can be observed in both the undifferentiated and differentiated cells (consistent with the differentiation effects in NOKs-Akata cells). This raises the question of whether low-level latent EBV infection in basal epithelial cells (too low to be reproducibly detected by EBER *in situ* hybridization staining) actually occurs as a normal component of EBV infection. If so, this would help to explain how EBV infection can lead to the undifferentiated form of nasopharyrngeal carcinoma. The relative paucity of lytic versus latent EBV transcripts in normal tonsil tissues of immunocompetent individuals is perhaps somewhat surprising, given how much lytic protein expression is observed in the differentiated epithelial cells of OHL lesions. We speculate that the innate and/or adaptive immune responses in immunocompetent individuals very efficiently inhibits viral reactivation and/or eliminates cells with lytic infection, and that latently infected cells are more resistant to the host immune responses. In the future, it will be important to examine oral epithelium from patients who have recently recovered from infectious mononucleosis, since such patients secrete very high levels of infectious viral particles in their saliva for up to one year after infection, and thus latent and lytic EBV transcripts (and proteins) may be easier to detect in the normal oropharyngeal cells of these individuals.

## Materials and Methods

### Ethics statement

The research using oral hairy leukoplakia lesions for IHC analysis in Fig 10 was approved by Institutional Review Board (IRB) of our institution (University of Wisconsin Madison), as well as the IRB of our collaborating institution (University of California, San Francisco). All adult subjects provided written informed consent (and no children were in the study). The oral hairy leukoplakia studies shown in Table 2, approved by the IRB of our collaborating institution (Louisiana State University Health Sciences Center, Shreveport), used de-identified tissues obtained from the AIDS and Cancer Resource Specimen resource. The tonsil studies shown in Table 3 and S6 Fig, approved by the IRB of our collaborating institution (Louisiana State University Health Sciences Center, Shreveport), used de-identified samples considered “left over” material by the pathologist that would have been discarded otherwise. The IHC studies on normal tongue and tonsil tissue shown in Figs 9 and S5 used de-identified tissues purchased commercially from Abcam and IHC World and were approved by the University of Wisconsin School of Medicine IRB.

### Cell lines and culture

The NOKs cell line (a gift from Karl Munger, Tufts University) is a telomerase immortalized normal oral keratinocyte cell line that was derived as previously described [85]. NOKs cells were cultured in keratinocyte serum free medium (KSFM) (Life technologies, Inc.) supplemented with epidermal growth factor and bovine pituitary extract. The NOKs-Akata cell line was derived by co-culturing the NOKs cells with Burkitt lymphoma cells containing the Akata strain of EBV (with an inserted G418 resistance selectable marker and a green fluorescent protein (GFP) gene) [86] and then selecting with 50 ug/ml G418, as previously described [36]. The Akata-GFP Burkitt lymphoma cell line was a gift from Kenzo Takada [received from Bill Sugden]). HONE-Akata cells (a gift from Lawrence Young, University of Birmingham) and CNE2-Akata cells (a gift from K.W. Lo at the Chinese University of Hong Kong [received via Diane Hayward]) are EBV-superinfected (Akata strain) HONE and CNE2 epithelial cell carcinoma cell lines that were originally thought to be derived from nasopharyngeal carcinomas but have been recently shown to be HPV infected and at least partially derived from HeLa cells [87]. Both of these cell lines were cultured in RPMI medium with 10% fetal bovine serum (FBS), 1% penicillin-streptomycin (pen-strep) and 400 μg/mL G418. C666-1 cells (a gift from Dolly Huang), an EBV-infected nasopharyngeal carcinoma line [88], and SNU.719 cells, a gastric carcinoma line harboring EBV [89], were cultured in RPMI with 10% FBS and 1% pen-strep. AGS gastric carcinoma cells **(**obtained from ATCC) were maintained in F-12 medium supplemented with 10% FBS and 1% pen-strep. AGS-Akata cell line is derived from AGS cells superinfected with Akata strain of EBV and selected for G418 resistance as previously described [90]. Raji (obtained from ATCC), Mutu I (a gift from Alan Rickinson), Mutu III, Jijoye (ATCC), and Kem I (a gift from Jeffrey Sample) are EBV-positive Burkitt lymphoma cell lines and were cultured in RPMI supplemented with 10% FBS and 1% pen-strep. D4 LCL is an EBV-transformed (B95.8) B cell lymphoblastoid cell line (LCL).

### Plasmids

Plasmid DNAs were purified using QIAGEN Plasmid Maxi Kits as described by the manufacturer. pCDNA3.1-HA- KLF4 (Addgene plasmid # 34593, a gift from Michael Ruppert) expresses an amino-terminal HA-tagged KLF4 [91]. pCDNA3-BLIMP1 expresses an amino-terminal FLAG-tagged BLIMP1 [92]. Plasmid pCpGL- Zp -668 is a luciferase reporter construct containing nucleotides -668 through +15 (relative to the transcription initiation site) of the EBV BZLF1 IE promoter (Zp) cloned between the SpeI and BglII restriction sites of pCpGL (a gift from Michael Rehli [93]), a CpG-free vector driving expression of the luciferase gene. The pCpGL-Zp-40 construct contains nucleotides -40 through +15 (relative to the transcription initiation site) of the EBV BZLF1 IE promoter and serves as a negative control in some experiments. Plasmid pCpGL-Rp-1068 is a luciferase reporter construct containing nucleotides -1068 through +38 (relative to the transcription initiation site) of EBV BRLF1 IE promoter (Rp) cloned between the SpeI and BglII restriction sites of pCpGL. The 5’ Rp deletion mutants were constructed as described previously [39]. The names of the 5’ promoter deletions indicate the number of promoter nucleotides present in each construct upstream of transcription start site. Site-directed Rp mutants altering KLF4 consensus sites were constructed in the pCpGL-Rp-551 vector using the Strategene QuikChange Site-Directed Mutagenesis Kit, as per the manufacturer’s protocol, using the following primers: Mutant 1–5’-CTCTGGACATCCGCACGAATCAAATCACAATTTTTGGAGACCCGTC- 3’ and Mutant 2–5’- GCCCGGAGCAATGACTCTAGTTTGTCCTTGTGTGAGGTC-3’.

### Methylation of reporter constructs

Reporter gene constructs were methylated *in vitro* using CpG methyltransferase M.SssI (New England Biolabs) as per the manufacturer’s protocol. Methylated and mock treated reporter gene constructs were cleaned by phenol chloroform extraction and salt precipitation, and complete methylation of these constructs was then confirmed by cutting the DNA with both HpaII (which cannot digest methylated DNA) and MspI (which cuts irrespective of the methylation state).

### Organotypic raft cultures

Transwell inserts (24 mm in diameter and 0.4 μm in pore size; Costar) were coated with 1 ml of collagen (3.0 mg/ml; Wako Chemicals) premix consisting of F-12 medium, 10% FBS and 1% Pen/Strep. Human foreskin fibroblasts (600 μl at 7.5 × 10^5^ cells/ml) were embedded into the remaining collagen mix and 2.5 ml was plated onto the collagen-coated Transwell inserts. The collagen-coated Transwell insert with embedded human fibroblasts was allowed to incubate for 4 days in a 5% CO_2_ incubator at 37°C in F-12 medium containing 10% FBS and 1% Pen/Strep. After 4 days, 150 μl of keratinocytes (1.4 x 10^6^ cells/ml) in keratinocyte plating medium (F medium [1.88 mM Ca2+]) containing 0.5% FBS, adenine (24 μg/ml), cholera toxin (8.4 ng/ml), hydrocortisone (2.4 μg/ml), and insulin (5 μg/ml) were plated onto the collagen dermal equivalent. Four days after plating, the Transwell inserts were placed onto three 1-in^2^ cotton pads (Bio-Rad) in a six well tray (BD Biosciences). The rafts were fed from below the Transwell insert with cornification medium (keratinocyte plating medium containing 5% FBS and 10 μM C8:0) every other day. Eleven days after being lifted to the liquid-air interface, the rafts were fed for 8 h with cornification medium containing 10 μM bromodeoxyuridine (BrdU). Subsequently, the rafts were embedded in 2% agar-1% formalin, fixed in 4% formalin at 4°C overnight, embedded in paraffin, and cut into 4-μm-thick cross sections.

### Chemical reagents

NOKs-Akata cells were treated for 48 hours with the following chemical reagents to induce lytic EBV reactivation: phorbol 12-myristate 13-acetate (TPA; 20 ng/ml; Sigma), sodium butyrate (3 mM; Sigma) and calcium chloride dehydrate (1.2 mM, Sigma). For ROCK inhibitor experiments, cells were treated with ROCK inhibitor Y27632 (10 μM, Enzo lifesciences) at the same time as the TPA treatment. B cells were treated with the following chemical reagents to induce lytic EBV reactivation: anti-human IgG (10 μg/ml; Sigma), 5-aza-2’-deoxycytidine (2μM; Acros Organics), and TGFβ (5 ng/ml; Biolegend).

### Immunofluorescence (IF) studies

Formalin-fixed, paraffin-embedded tissue sections were deparaffinized and then examined by IF as previously described [94]. Primary antibodies used were anti-Z (BZ.1) monoclonal antibody (1:200, Santa Cruz Biotechnology SC-53904), anti-K10 polyclonal antibody (1:1,000, Covance PRB-159P), and anti-involucrin (SY5) monoclonal antibody (1:1000, Sigma, I9018). Secondary antibodies used were Alexa 594 conjugated goat anti- rabbit (red) (Invitrogen A-31571) and Alexa 488 conjugated goat anti-mouse (green) (Invitrogen 21206).

### Immunohistochemistry (IHC) and EBER studies

Formalin-fixed, paraffin-embedded tissue sections were deparaffinized and then examined by IHC as previously described [95]. Antibodies used included anti-Z (BZ.1) monoclonal antibody (1:200, Santa Cruz Biotechnology SC-53904), anti-BMRF1 monoclonal antibody (1:200, Vector Laboratories VP-E608), anti-KLF4 polyclonal antibody (1:500, Sigma-Aldrich HPA002926) and anti-BLIMP1 (1:1000, Sigma-Aldrich HPA030033). Human normal tongue tissue and tonsil tissue slides were commercially purchased (Abcam and IHC World). EBER *in situ* hybridization studies were performed using the PNA ISH Detection Kit (DakoCytomation) according to the manufacturer’s protocol as previously described [95].

### Fluorescence in situ hybridization (FISH)

Formalin-fixed, paraffin-embedded tissue sections were deparaffinized and then examined by FISH. A digoxigenin (DIG-11-dUTP, Roche)-labeled probe was used to analyze viral DNA amplification. Nick translation was used to label EBV bacmid DNA (B95.8) with digoxigenin to make the probe. Deparaffinized sections were incubated in pre-hybridization buffer (2XSCC, 0.5% IPECAL, pH 7.0) for 30 minutes at 37°C. Sections were dehydrated using a series of ice cold ethanols (70%, 80%, 95%) for 2 minutes each. Sections were dried by placing them in an empty container at 50°C for 5 minutes. Sections were then placed in denaturation solution (28 mL formamide, 4 mL 20X SSC pH 5.3, 8 mL water) at 72°C for 2 minutes. The ethanol series was repeated again, and after drying the sections, denatured probe was added to the slides. The probe was hybridized to the raft sections overnight at 37°C in a humidified chamber. After washing for 30 minutes twice with 2XSSC and 50% formamide at 50°C and 30 minutes twice with 2XSSC at 50°C, signals were detected with a digoxigenin-specific antibody conjugated to fluorescein isothiocyanate (Sigma, F3523) at 2% by volume in STM solution (4X SSC, 5% non-fat dried milk, 0.05% Tween-20, 0.002% sodium azide) for 30 minutes at 37°C. Nuclei were counterstained with DAPI.

### Transient transfections

Plasmid DNA was transfected into epithelial cells with Lipofectamine 2000 transfection reagent (Invitrogen) according to the manufacturer’s protocol. In general, epithelial cells (in a 12 well plate) were transfected with either 100 ng pcDNA3-KLF4, 100 ng pcDNA3-BLIMP1, or 50 ng pcDNA1-KLF4 plus 50 ng pcDNA3-BLIMP1, in addition to 400 ng pcDNA3.1. KLF4 siRNA (Origene SR306162) was transfected into epithelial cells with Lipofectamine RNAiMax (Invitrogen), as per the manufacturer’s protocol. Raji and Jijoye cells were transfected using Amaxa cell line nucleofactor kit V, as per the manufacturer’s protocol.

### Immunoblot analysis

Cell lysates were harvested in Sumo lysis buffer including protease inhibitors (Roche) as described previously [96]. Protein concentration was determined using the Sumo protein assay (Biorad), and proteins were separated in SDS-10% polyacrylamide gels and then transferred onto a nitro-cellulose membrane. Membranes were blocked in PBS containing 5% milk, and 0.1% Tween 20 solution. Membranes were then incubated in the following primary antibodies: anti-Z (Santa Cruz, product # sc-53904, 1:250), anti-BMRF1 (Millipore, product # MAB8186, 1:3,000), anti-R rabbit polyclonal antibody directed against the R peptide (peptide sequence EDPDEETSQAVKALREMAD, 1:2,500), anti-KLF4 (Cell Signaling, product # 4038, 1:1,000), anti-BLIMP1 (Cell Signaling, product # 9115, 1:1,000), anti-β-actin (Sigma, product # A5441,1:5,000), anti-tubulin (Sigma, product # T5168, 1:2,000), and anti-involucrin (Sigma, product # I9018, 1:3000). The secondary antibodies used were horseradish peroxidase (HRP)- labelled goat anti-mouse antibody (Fisher Scientific, 1:5,000) and HRP- labeled anti-rabbit antibody (Fisher scientific, 1:5,000).

### Reporter gene assays

Cells were washed with cold PBS and harvested 48 hours after transfection in 1X reporter lysis buffer (Promega), subjected to one freeze-thaw cycle, and then the relative luciferase units were quantified using a BD Monolight 3010 luminometer (BD Biosciences) and luciferase assay reagent (Promega). The fold change for each condition was calculated relative to the promoter activity in the presence of the control vector, pCDNA. For each condition, at least 3 independent experiments were performed in duplicates.

### Chromatin-immunoprecipitation and quantitative PCR (ChIP-qPCR) assays

2x10^7^ cells were cross-linked in 1% (w/v) formaldehyde (Sigma) for 5 min at room temperature and the cross-linking reaction was quenched by addition of glycine to a final concentration of 0.125M. Cells were washed twice with cold PBS and lysed in 1 ml of lysis buffer (50 mM Tris-HCl [pH 8.1], 10 mM EDTA, 1% [w/v] SDS, 1 mM PMSF, 1 μg/ml leupeptin, 20 μg/ml aprotinin) for 30 min on ice before extensive sonication with a Qsonica LLC Q700 sonicator. After extract clearing by centrifugation, supernatants were diluted 1:10 in dilution buffer (16.7 mM Tris-HCl [pH 8.1], 1.2 mM EDTA, 167 mM NaCl, 1.1% [v/v] Triton X-100, 0.01% [w/v] SDS, 1 mM PMSF, 1 μg/ml leupeptin, 20 μg/ml aprotinin). Aliquots of each input chromatin lysate were reserved for qPCR analysis. 1ml of diluted chromatin lysate was incubated with ChIP antibodies with rotation at 4°C overnight. Primary antibodies used were anti-KLF4 polyclonal antibody (H-180, Santa Cruz 20691) and anti-RNA polymerase II S5 phospho-specific antibody (4H8, Abcam ab5408). 15ul Protein A/G magnetic beads (Thermo 88802) were added to each 1ml ChIP and incubated for 1 hour at 4°C with rotation. Next, magnetic beads were pelleted with magnetic separation rack and washed once with cold low salt wash buffer (20 mM Tris-HCl [pH8.1], 2 mM EDTA, 150 mM NaCl, 1% [v/v] Triton X-100, 0.1% [w/v] SDS), once with high salt wash buffer (identical to low salt wash buffer, except 500 mM NaCl), once with LiCl wash buffer (10 mM Tris-HCl [pH8.1], 1 mM EDTA, 0.25 M LiCl, 1% [v/v] NP40, 1% Deoxycholic acid), and finally twice with TE buffer (10 mM Tris-HCl [pH8.1], 1 mM EDTA). Samples were then resuspended in 150 μl of elution buffer (0.1 M NaHCO_3_, 1% [w/v] SDS) and rotated for 20 min at room temperature. Two rounds of elution of protein-DNA complexes were pooled. Reversal of cross-linking was accomplished by incubation of pooled eluates at 65°C for 4 hours after addition of NaCl to final concentration of 200mM and 100ug/ml Proteinase K. DNA was purified using the QIAquick PCR purification kit (28706; Qiagen) and quantified using a BioRad CFX96 system with the iTaq universal SYBR Green supermix (1725121;Bio-Rad). Purified input chromatin lysate was used in real-time PCR reactions for standardization.

### Viral titer assay

Viral titration assays were performed in CNE2-Akata cells, as previously described [97]. CNE2-Akata cells were transfected with either control vector, KLF4, BLIMP1 or KLF4 and BLIMP1 together (for synergy studies). Supernatant was harvested, 96 hours after transfection, and was passed through a 0.8 um filter. 10 uL of this supernatant (for each condition) was used to infect Raji cells (2 x 10^5^ cells/ condition), followed by the addition of phorbol-12-myristate-13-acetate (TPA) (20 ng/ml) and sodium butyrate (3 mM final concentration), 24 hours after infection. Viral titer was determined by counting the number of GFP-positive Raji cells, 48 hours after infection.

### Viral DNA replication quantitative PCR assay

HONE-Akata cells were transfected with either control vector, KLF4, BLIMP1or KLF4 and BLIMP1 together. Intracellular DNA was harvested from these cells, 96 hours after transfection, using the GenElute mammalian genomic DNA miniprep kit (Sigma) according to manufacturer’s protocol. Primers directed against the EBV BZLF1 promoter (forward primer – 5’-TGCCTGTGGCTCATGCATAGTTTC-3’ and reverse primer – 5’-GCCATGCATATTTCAACTGGGCTG–3’) were used to quantify viral DNA. DNA was also amplified using primers directed against the beta-globin gene (forward primer – 5’-GAGGCTCTGACCATAACCAAA-3’ and reverse primer- 5’-GACAAGGCTGCAAGCTATACTA-3’), and the EBV quantification was normalized to the beta-globin result to correct for variations in DNA quality and quantity. All samples were assayed in duplicate. DNA was amplified using the iTaq universal SYBR Green supermix as suggested by the manufacturer (catalogue # 1725121; Bio-Rad) in a BioRad CFX96 machine. The PCR amplification protocol was initiated at 98°C for 2 minutes followed by 39 PCR cycles consisting of 5 seconds at 98°C followed by 60°C for 30 seconds.

### KLF4 mutagenesis using CRISPR-Cas9 technology

Mutagenesis of KLF4 was performed using the CRISPR-Cas9 technology, as previously described [98,99]. The following oligos were annealed and cloned into LentiCRISPRV.2 plasmid (Addgene plasmid # 52961, gift from Feng Zheng) [98]: Oligo 1: 5’-CACCGGGAGCCGGTGCGGCTTGCGG-3’ and Oligo 2–5’-AAACCCGCAAGCCGCACCGGCTCCC-3’. 4 ug of either the control LentiCRISPRV.2 plasmid or plasmid containing the KLF4 guide RNA, 0.6 ug VSV-G and 1.4 ug of ps-Pax2 (a gift from Didier Trono [Addgene plasmid # 12260]) were co-transfected into 293T cells in a 10 cm dish to package the lentivirus. Supernatant, containing the lentivirus, was harvested at 2 and 3 days post infection and was used to infect HONE-Akata cells. Infected HONE-Akata cells were selected using 1 ug/mL of puromycin. KLF4 mutagenesis (knock out) was confirmed using western blot analysis.

### Laser capture microdissection

Paraffin-embedded specimens were sectioned to a thickness of 4–5 μm, stained with hematoxylin and eosin and kept in desiccant until used for laser capture microdissection (LCM). Institutional Review Board approval was obtained. The cells of interest were identified by their morphology and captured by a PixCell IIe LCM system (Arcturus Engineering, Inc., Mountain View, CA, USA). CapSure HS LCM caps (Arcturus) coated with infrared light absorbing ethylene vinyl acetate (EVA) were placed over the tissue. The laser spot size and power were adjusted to melt the EVA film and capture cells only in the area irradiated by the very low-power infrared targeting beam. The laser power was 55–60 mW, laser pulse duration was 1.5–1.8 msec, and laser spot size was 7.5 μm in diameter. Total RNA was extracted, purified, DNase treated, and reverse transcribed from the LCM captured cells using the Paradise Whole Transcript RT Reagent System (Arcturus) according to the manufacturer’s instructions. A human formalin-fixed universal reference RNA was reverse transcribed in parallel for use as a positive control.

### Real-time quantitative PCR

Real-time quantitative PCR (QPCR) was performed using an ABI Prism 7000 Sequence Detector with SYBR Green. The PCR reactions were set up in a 96-well optical plate in duplicate by adding the following reagents into each well: 2.5 μl of cDNA, 12.5 μl of SYBR Green PCR Master Mix (Applied Biosystems, Foster City, CA, USA); the final concentrations of primers were 0.3 μmol/L in a final volume of 25 μl. The PCR amplification protocol was initiated at 50°C for 2 minutes followed by 10 minutes at 95°C and 40 PCR cycles consisting of 15 seconds at 95°C followed by 60°C for 1 minute. To exclude the possibility of contamination with genomic DNA each reaction also contained a control PCR amplification of isolated RNA to which no reverse transcriptase had been added and a water only control. The sequences of the primers that were used are summarized in Table 1. All samples were tested with the reference genes glyceraldehyde-3-phosphate dehydrogenase (GAPDH) or Cyclophilin A (CycA) for data normalization to correct for variations in RNA quality and quantity. All samples were assayed in duplicate or triplicate, and values were expressed as mean ± standard deviation. Control experiments with freshly isolated peripheral B cells admixed with cultured epithelial cells showed that the sensitivity of the assay was such that 1 B cell could be detected in a background of 1,000 epithelial cells with threshold cycle (Ct) values for GAPDH and CD20 of 26.40±0.16 and 38.78±0.18 respectively. The specificity of amplification of targets with high Ct values was confirmed by analysis of the temperature dissociation curves.

## Supporting Information

S1 Fig Uninfected NOKs cells have no EBER or lytic EBV antigen expression. Uninfected (left panel) and EBV-infected (right panel) NOKs cells were grown in organotypic air-interface raft culture, and *in situ* hybridization or immunohistochemistry was performed to detect expression of the EBV EBERs or lytic EBV proteins (Z and BMRF1) as indicated. Arrows indicate examples of Z and BMRF1 positive cells in the EBV-infected line.
(TIF)

S2 Fig EBV lytic DNA amplification occurs in suprabasal layers of EBV-infected NOKs organotypic raft cultures. Uninfected NOKs (upper panel) and NOKs-Akata (lower panel) cells were grown in organotypic raft culture, formalin fixed, embedded in paraffin and 5 micron thick sections were analyzed by fluorescence in situ hybridization (FISH) analysis using an EBV-specific probe (green). Blue nuclear counterstain is DAPI. While small green foci representing latent EBV genomes are present in every cell (only detectable at higher magnification), this low magnification image shows an example of a rare cell containing amplified EBV DNA in the suprabasal layers of the raft represented by intense green signal filling the nucleus.
(TIF)

S3 Fig KLF4 binds to the Rp IE EBV promoter, and enhances its association with activated RNA polymerase II, in NOKs-Akata cells. NOKs-Akata cells were transfected with either control vector or a KLF4 expression vector, and ChIP assay was performed 48 hours after transfection. Cross-linked DNA-protein complexes were immunoprecipitated using anti-KLF4 antibody (top panel), or anti-phospho-RNA polymerase II antibody (bottom panel) and control IgG antibody in each case. Quantitative PCR was performed to quantitate the amount of DNA pulled down for the IE Rp (left panel), and negative control Cp (right panel) EBV promoters.
(TIF)

S4 Fig KLF4 synergizes with BLIMP1 to induce EBV late gene expression and lytic replication in latently infected epithelial cells. Control vector or KLF4 and BLIMP1 expression vectors (either alone or in combination) were transfected into A) HONE-Akata cells, B) NOKs-Akata cells, or C) SNU.719 gastric carcinoma cells and immunoblot analysis was performed to compare the levels of transfected KLF4 and BLIMP1, and induction of EBV late viral capsid protein, p18. Tubulin or Actin served as a loading control. D). Intracellular DNA was quantitated by qPCR analysis in HONE-Akata cells transfected with vector alone, KLF4 alone, BLIMP1 alone or the combination of KLF4 and BLIMP1. The level of intracellular EBV DNA is shown relative to the amount in the vector transfected cells and has been plotted as mean +/- standard deviation.
(TIF)

S5 Fig KLF4 and BLIMP1 expression is induced by differentiation in tonsil epithelial cells. H&E analysis, and immunohistochemistry analysis was performed on a paraffin-embedded, formalin-fixed biopsy of normal tonsil tissue using antibodies directed against KLF4 and BLIMP1 as indicated (Images: 40x).
(TIF)

S6 Fig EBER-positive staining of B cells and epithelial cells in normal tonsil tissue. Examples of EBER staining of B cells (upper panels), and epithelium (lower panels) within tonsil tissues that were used to obtain the data shown in Table 3 are shown.
(TIF)

S7 Fig Treatment with lytic inducing agents does not restore KLF4 expression in Burkitt lymphoma cells. Akata Burkitt lymphoma cells, treated with or without anti-IgG or 5-Aza-2’-deoxycytidine, or Mutu I cells treated with or without TGF beta, were analyzed by immunoblot analysis to detect the expression of lytic viral proteins, Z and BMRF1, and cellular proteins, KLF4 and GAPDH (a loading control). NOKs cells served as a positive control for KLF4 expression. The type and duration of each treatment is indicated above each lane.
(TIF)

S8 Fig KLF4 induces lytic EBV gene expression in Jijoye Burkitt lymphoma cells. Jijoye cells were transfected with either control vector or a KLF4 expression vector and immunoblot analysis was performed to compare the levels of transfected KLF4 and lytic viral proteins Z, and BMRF1. Actin served as a loading control.
(TIF)
