# Supplementary figures and images for: Differentiation-Dependent KLF4 Expression Promotes Lytic Epstein-Barr Virus Infection in Epithelial Cells
Source: PLoS Pathog. 2015 Oct 2;11(10):e1005195. doi: 10.1371/journal.ppat.1005195 (PMC4592227; doi:10.1371/journal.ppat.1005195)

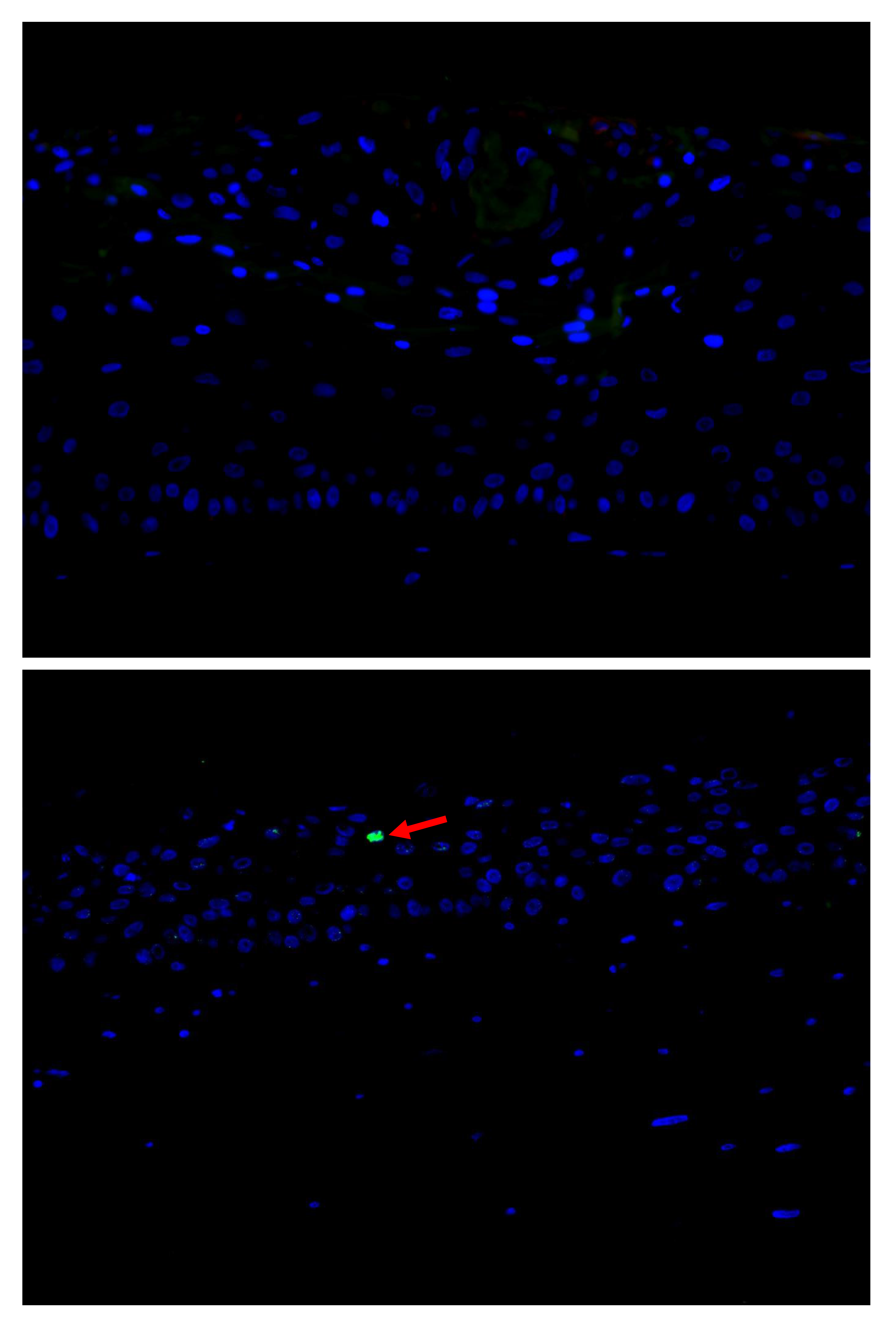

Supplement: S2 Fig — Uninfected NOKs (upper panel) and NOKs-Akata (lower panel) cells were grown in organotypic raft culture, formalin fixed, embedded in paraffin and 5 micron thick sections were analyzed by fluorescence in situ hybridization (FISH) analysis using an EBV-specific probe (green). Blue nuclear counterstain is DAPI. While small green foci representing latent EBV genomes are present in every cell (only detectable at higher magnification), this low magnification image shows an example of a rare cell containing amplified EBV DNA in the suprabasal layers of the raft represented by intense green signal filling the nucleus. (TIF) [file ppat.1005195.s002.tif]

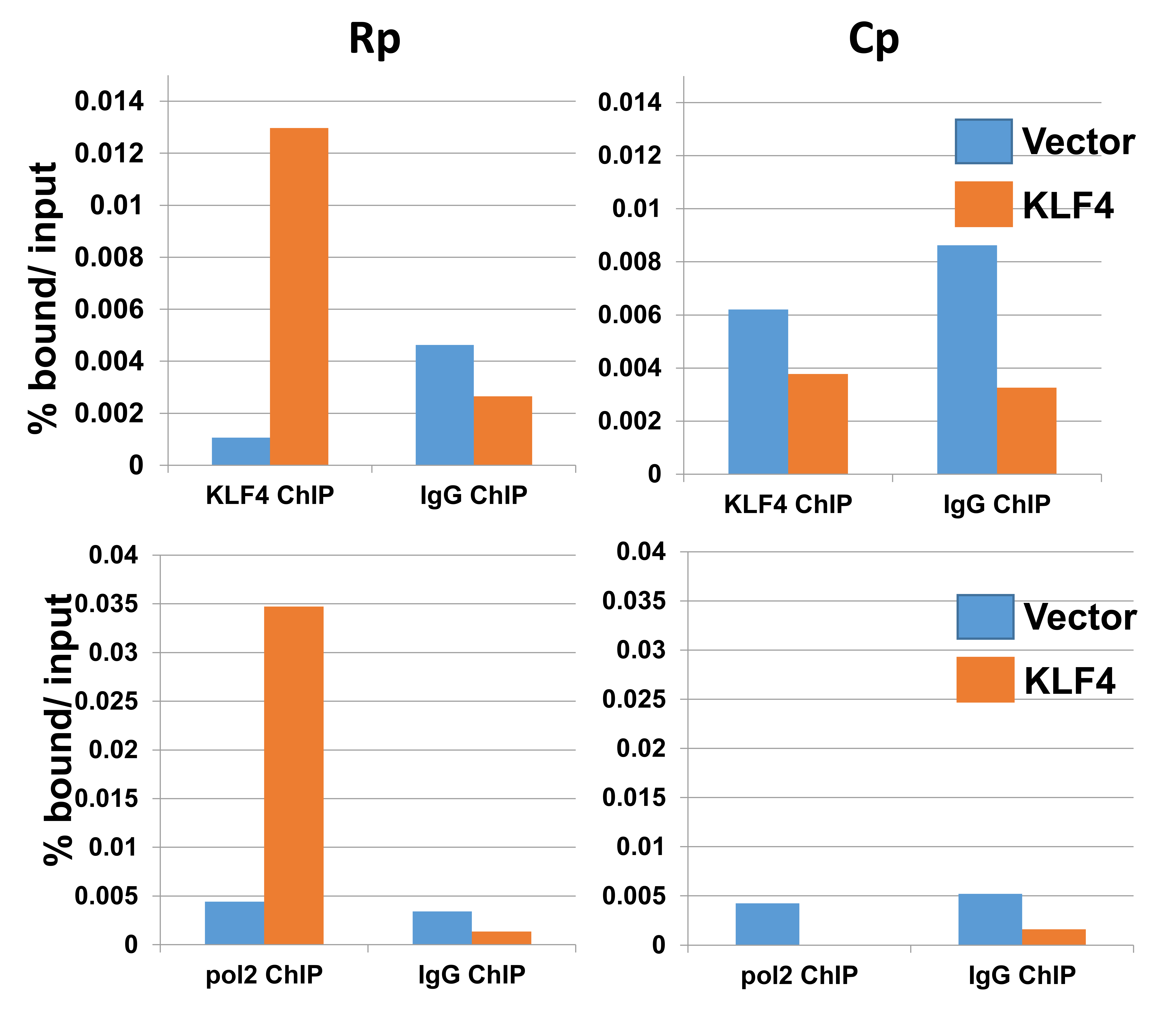

Supplement: S3 Fig — NOKs-Akata cells were transfected with either control vector or a KLF4 expression vector, and ChIP assay was performed 48 hours after transfection. Cross-linked DNA-protein complexes were immunoprecipitated using anti-KLF4 antibody (top panel), or anti-phospho-RNA polymerase II antibody (bottom panel) and control IgG antibody in each case. Quantitative PCR was performed to quantitate the amount of DNA pulled down for the IE Rp (left panel), and negative control Cp (right panel) EBV promoters. (TIF) [file ppat.1005195.s003.tif]

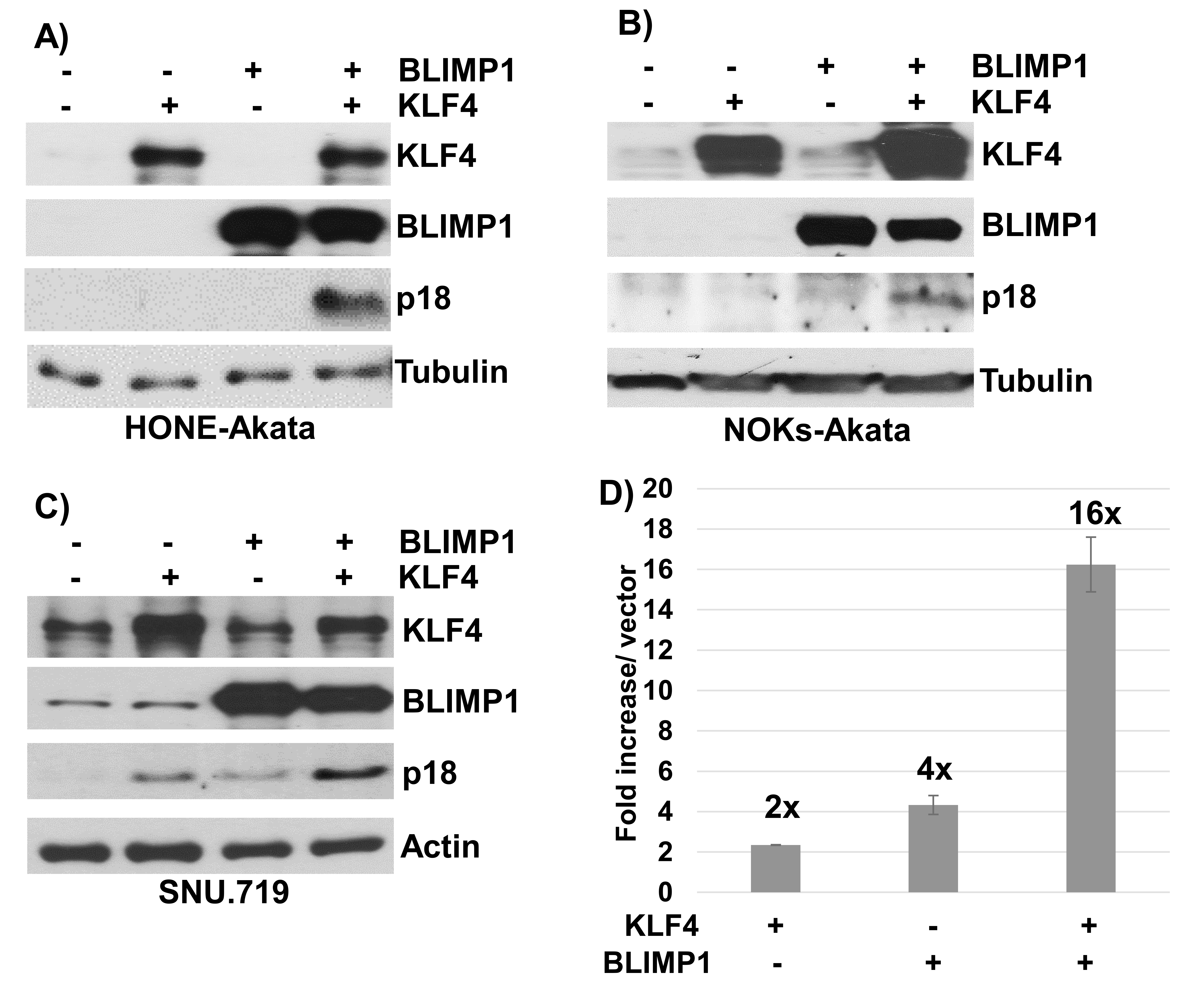

Supplement: S4 Fig — Control vector or KLF4 and BLIMP1 expression vectors (either alone or in combination) were transfected into A) HONE-Akata cells, B) NOKs-Akata cells, or C) SNU.719 gastric carcinoma cells and immunoblot analysis was performed to compare the levels of transfected KLF4 and BLIMP1, and induction of EBV late viral capsid protein, p18. Tubulin or Actin served as a loading control. D). Intracellular DNA was quantitated by qPCR analysis in HONE-Akata cells transfected with vector alone, KLF4 alone, BLIMP1 alone or the combination of KLF4 and BLIMP1. The level of intracellular EBV DNA is shown relative to the amount in the vector transfected cells and has been plotted as mean +/- standard deviation. (TIF) [file ppat.1005195.s004.tif]

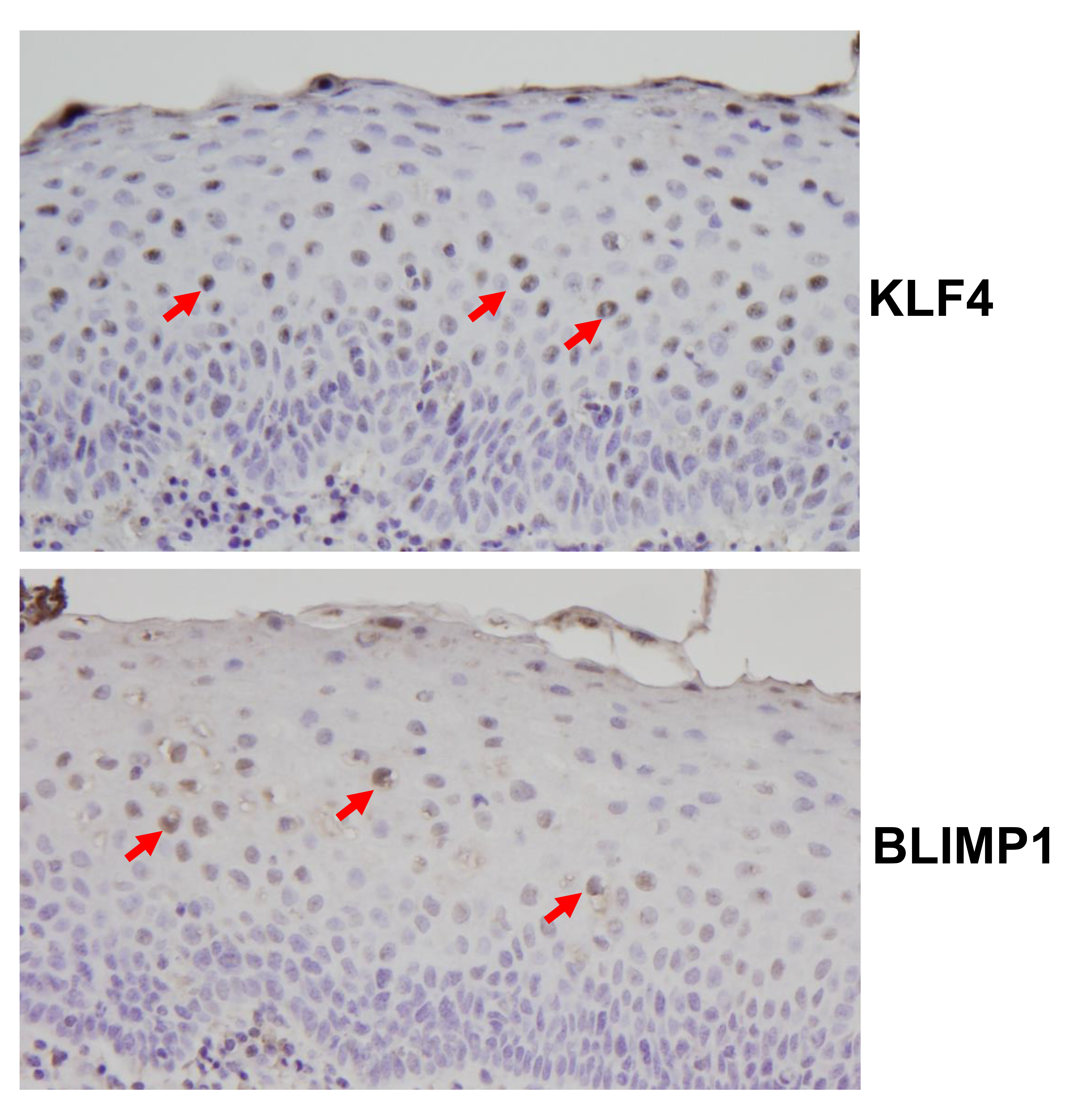

Supplement: S5 Fig — H&E analysis, and immunohistochemistry analysis was performed on a paraffin-embedded, formalin-fixed biopsy of normal tonsil tissue using antibodies directed against KLF4 and BLIMP1 as indicated (Images: 40x). (TIF) [file ppat.1005195.s005.tif]

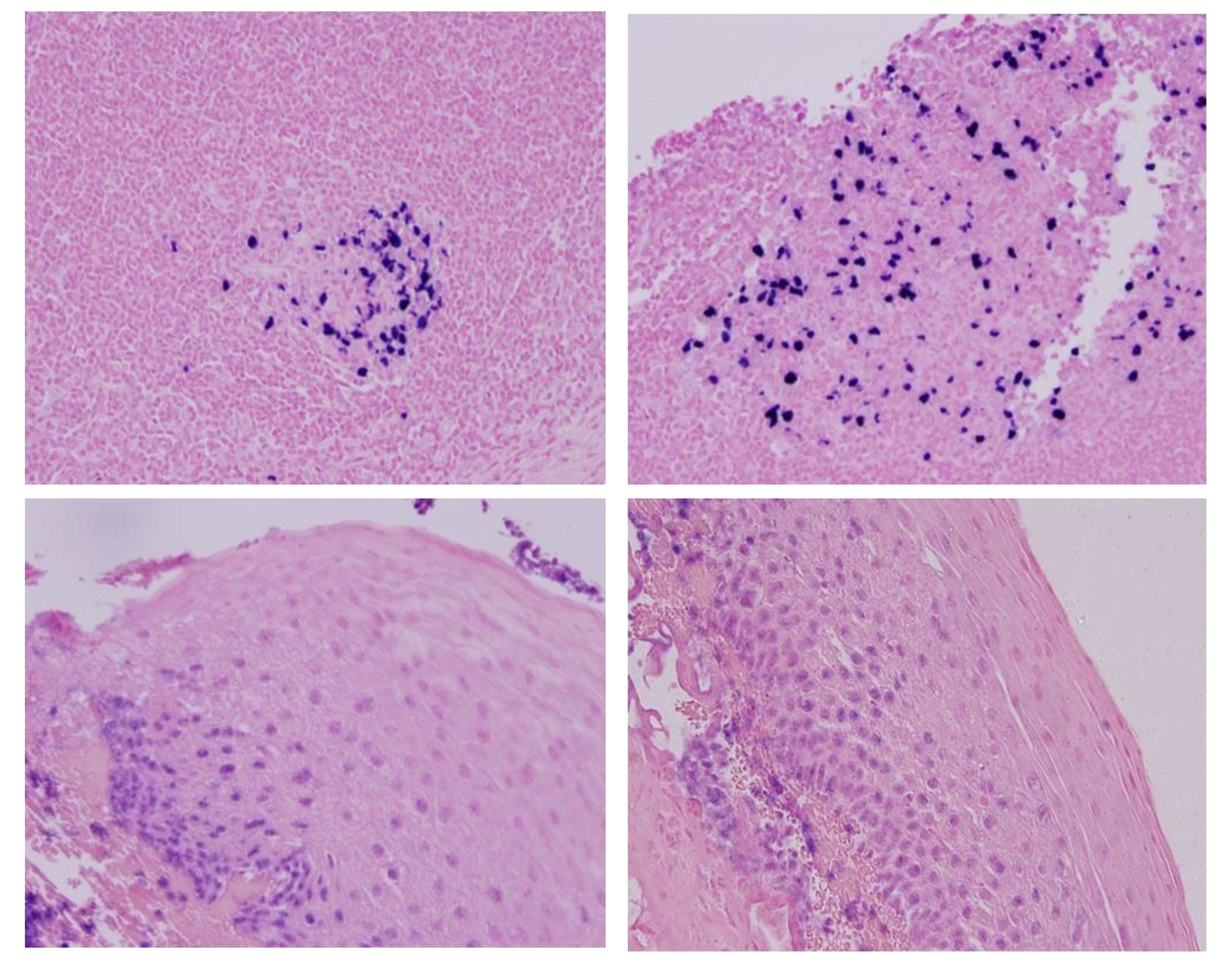

Supplement: S6 Fig — Examples of EBER staining of B cells (upper panels), and epithelium (lower panels) within tonsil tissues that were used to obtain the data shown in Table 3 are shown. (TIF) [file ppat.1005195.s006.tif]

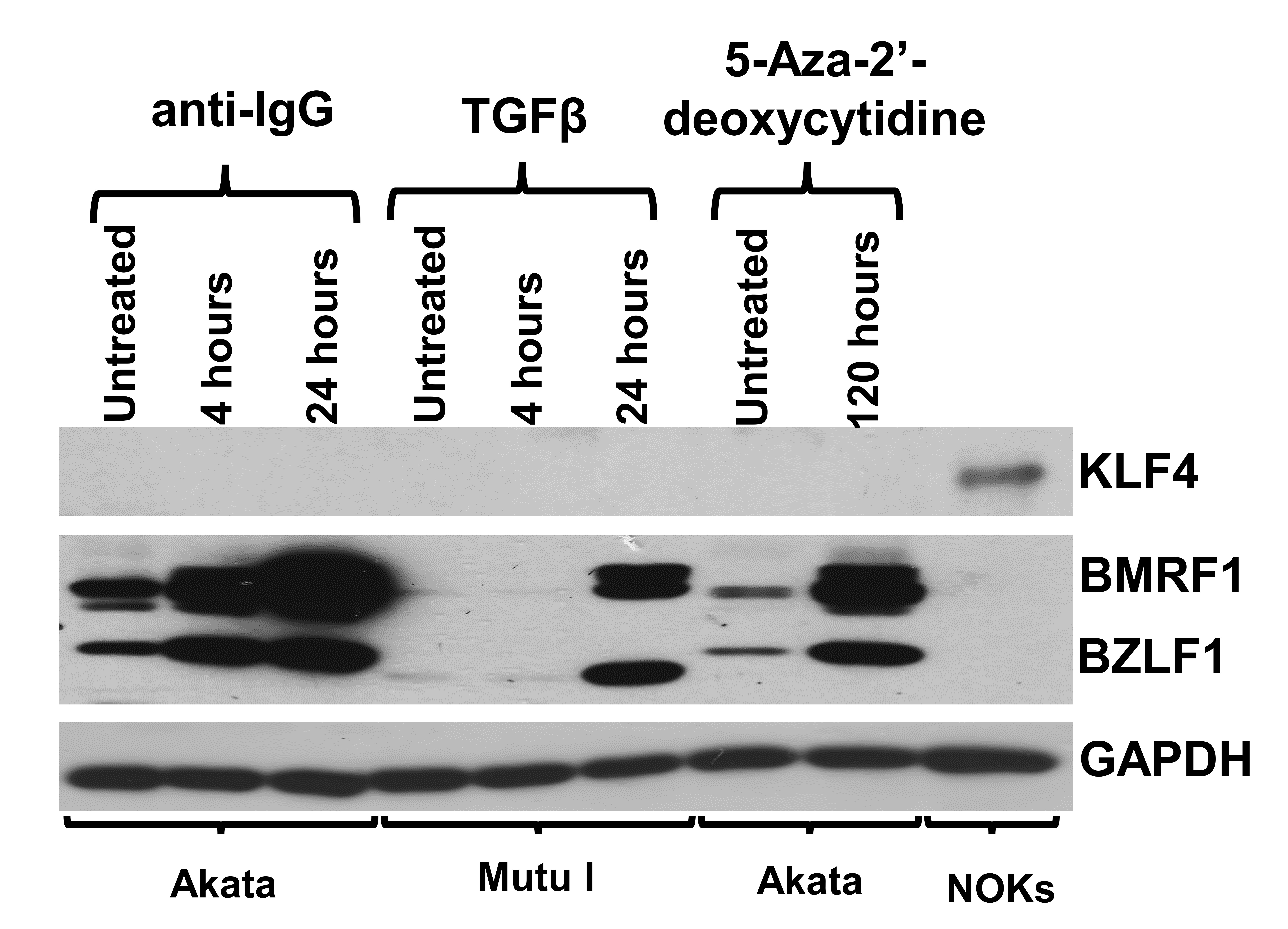

Supplement: S7 Fig — Akata Burkitt lymphoma cells, treated with or without anti-IgG or 5-Aza-2’-deoxycytidine, or Mutu I cells treated with or without TGF beta, were analyzed by immunoblot analysis to detect the expression of lytic viral proteins, Z and BMRF1, and cellular proteins, KLF4 and GAPDH (a loading control). NOKs cells served as a positive control for KLF4 expression. The type and duration of each treatment is indicated above each lane. (TIF) [file ppat.1005195.s007.tif]

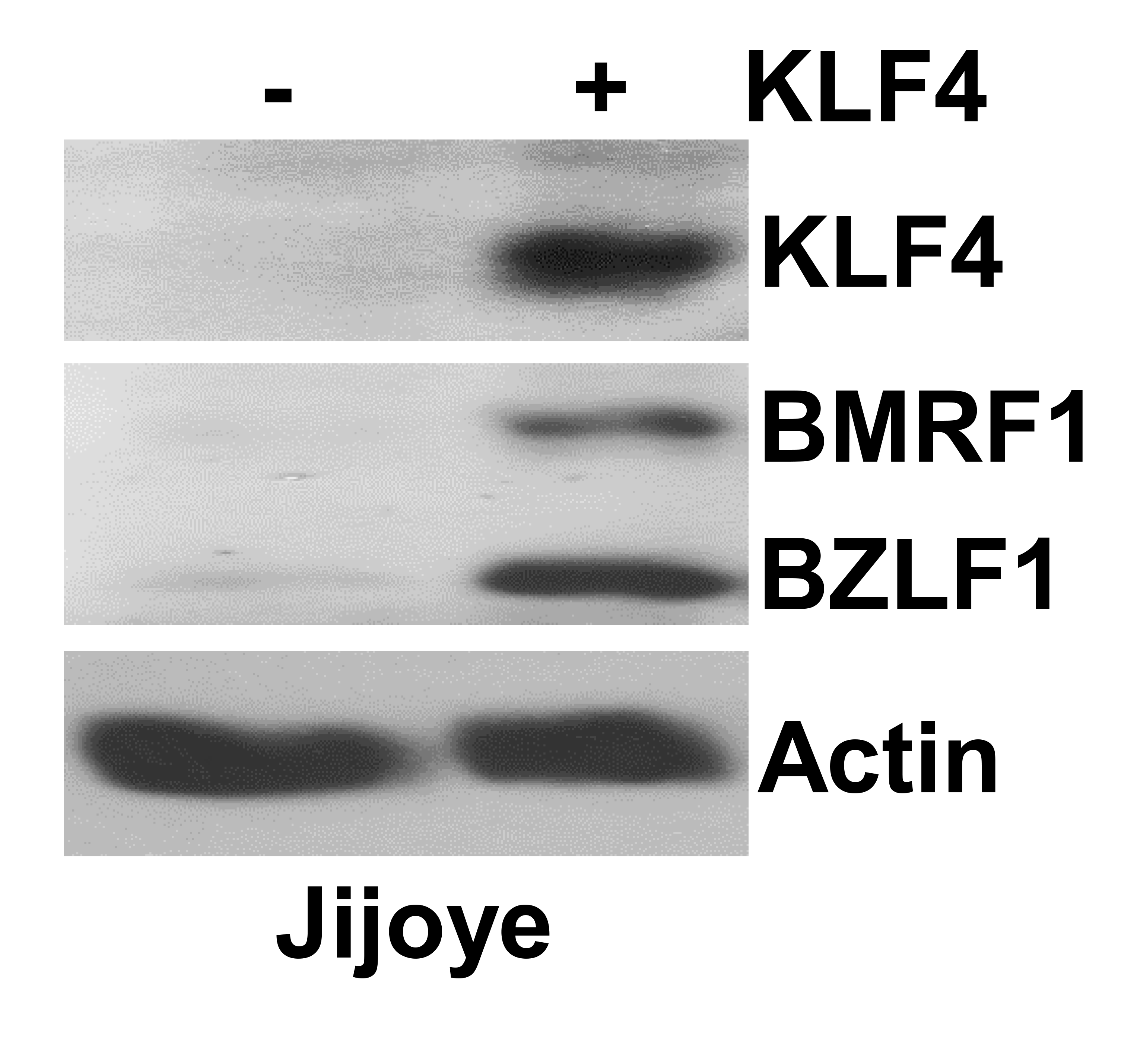

Supplement: S8 Fig — Jijoye cells were transfected with either control vector or a KLF4 expression vector and immunoblot analysis was performed to compare the levels of transfected KLF4 and lytic viral proteins Z, and BMRF1. Actin served as a loading control. (TIF) [file ppat.1005195.s008.tif]
